# Innovative myopic screening platform based on smartphones

**DOI:** 10.3389/fbioe.2025.1678800

**Published:** 2025-10-20

**Authors:** Qiang Su, Yicheng Ge, Jinghui Wang, Nan Jin, Haochen Han, Yingxin Li, Chea-Su Kee, Bei Du, Ruihua Wei

**Affiliations:** ^1^ Tianjin Key Laboratory of Retinal Functions and Diseases, Tianjin Branch of National Clinical Research Center for Ocular Disease, Eye Institute and School of Optometry, Tianjin Medical University Eye Hospital, Tianjin, China; ^2^ Department of Ophthalmology, Shanghai Key Laboratory of Orbital Diseases and Ocular Oncology, Shanghai Ninth People’s Hospital, Shanghai Jiao Tong University School of Medicine, Shanghai, China; ^3^ Chinese Academy of Medical Sciences and Peking Union Medical College, Institute of Biomedical Engineering, Beijing, China; ^4^ School of Optometry, The Hong Kong Polytechnic University, Hong Kong, China

**Keywords:** myopia, vision screening, subjective refraction, far point, smartphone measurement

## Abstract

**Introduction:**

To validate a novel smartphone-based approach for subjective refraction, specifically for myopia screening, offering a cost-effective and accessible tool for at-home vision assessment.

**Methods:**

A total of 230 healthy volunteers, encompassing 460 eyes, aged between 7 and 40 years (mean ± SD: 21.04 ± 7.76), and exhibiting refractive error (RE) ranging from −6.25 to +0.50 diopter (D), were deemed eligible subjects in this research. Subjective refraction assessments were conducted on all subjects, involving both the conventional phoropter examination by experienced optometrists which served as the clinical gold-standard, and the smartphone-based methodology. During smartphone measurement, the screen was gradually moved toward the eye until achieving clear differentiation of the ‘E’ optotypes and the astigmatic dial. The eye-to-smartphone screen distances (ESD) were calculated based on the image of iris diameter (ID) acquired by the front-facing camera of the smartphone. Applying the definitions of the far point and the rule of thirty, this allowed for the computation of sphere (S), cylinder (C), and astigmatism axis (*α*) values. The concordance between the two methods was assessed by establishing the Limits of Agreement (LOAs), which were calculated as the mean difference ± 1.96 times the standard deviation of the differences.

**Results:**

The smartphone-based screening technique showed that it closely matched the gold-standard subjective refraction used in clinical practice. The LOAs for S, C, and spherical equivalent refraction (SER) were 0.11 ± 0.89 D, −0.03 ± 0.82 D, and 0.10 ± 0.89 D, respectively. The average deviation in measuring the *α* was 4.72°, with 64.35% of deviations falling within the ±15° range. Additionally, the technique demonstrated impressive areas under the receiver operating characteristic curve (AUC) of 0.973 for the range of −3.00 D < SER ≤ 0.00 D and 0.986 for the range of −6.00 D < SER ≤ −3.00 D.

**Discussion:**

The innovative vision screening through smartphones can expand access to measuring RE, especially in home. The study confirms the validity of an innovative vision-screening approach for myopia. Its effectiveness and accessibility make it a valuable tool for opportunistic or large-scale myopia screening programs.

## 1 Introduction

Myopia represents a significantly prevalent global health concern (Shan et al., 2022). Presently, worldwide myopia prevalence surpasses 28.3%, with projections indicating a substantial increase to 49.8% by the year 2050. Particularly of concern is the escalating prevalence of high myopia, poised to rise from its current 4.0%–9.8% (Holden et al., 2016; Baird et al., 2020). Specifically, developing countries are grappling with the most significant burden of visual impairment. One of the principal contributors to this epidemic is the severe shortage of eye health professionals, compounded by the inequitable distribution of eye health services (Olusanya et al., 2016).

Additionally, the myopia rate of children is alarmingly high and continuing to rise in primary and secondary schools in China (Li et al., 2018; Choy et al., 2020; Thorn et al., 2021; Wang et al., 2021; Li et al., 2022; Yue et al., 2022). Refractive screening plays a pivotal role in myopia prevention and control (Loh and Chiang, 2018; Atowa et al., 2019; Crippa et al., 2022). However, the conventional approach to refractive screening incurs significant costs in terms of time, labor, equipment and management (Rodriguez-Lopez and Dorronsoro, 2022). Presently, the evaluation of eye refractive parameters is confined to eye hospitals or specialized optometry centers (Crippa et al., 2022), requiring families to invest significant time and resources in scheduling, travel, and clinic visits. Numerous children miss out on optimal opportunities for myopia correction, thereby significantly impeding myopia control efforts in China (Cui et al., 2021; Crippa et al., 2022). These challenges need to be addressed by the development of a cost-effective, precise, and user-friendly approach for home-based refraction.

Smartphones present a promising solution to these challenges. They are increasingly deployed as portable tools for scientific measurement, such as environmental monitoring and personal exposure assessment (Qu et al., 2023; Maanaki et al., 2025).

At present, several commercial technologies exist for measuring refractive error (RE) and visual acuity (VA). For the measurement of RE, the BV1000 by Topcon Corporation (Tokyo, Japan) (Dave and Fukuma, 2004) and the VAO by Voptica (Murcia, Spain) (Hervella et al., 2019) utilize automatic refraction and wavefront aberration technology with the limits of agreement (LOAs) of 0.05 ± 0.69 D and 0.00 ± 0.67 D in spherical equivalent refraction (SER), respectively. However, due to the professional requirement of skill and knowledge in operation, its applicability is restricted to hospital scenarios. The Easee BV (Amsterdam, Netherlands) (Wisse et al., 2019) and the Eyenetra (Cambridge, United States) (Tousignant et al., 2020) can be employed at home through smartphones or computer screens, which respectively exhibit LOAs of 0.13 ± 1.22 D and 0.53 ± 1.40 D for SER. The Eyenetra system requires a physical device attached to the smartphone, whereas the Easee tool is primarily used with a smartphone. There are several smartphone-based instruments for measuring VA, such as EyeHandBook (LOAs: 0.09 ± 0.17) (Tofigh et al., 2015), V@home (LOAs: 0.01 ± 0.24) (Han et al., 2019), Peek Acuity (LOAs: 0.07 ± 0.41) (Bastawrous et al., 2015) and McAndrew (Intraclass correlation coefficient, ICC = 0.917) (O'Neill and McAndrew, 2016). Despite being user-friendly, these refractive screening methods typically assess only VA and lack the ability to quantitatively analyze RE. Salmerón-Campillo (Salmeron-Campillo et al., 2024) leveraged longitudinal chromatic aberration (LCA) via blue OLED screens, reporting sphere (S) error of LOAs = 0.08 ± 0.67 D against clinical standards. Salmerón-Campillo recently expanded their method to measure sphero-cylindrical over-refraction, achieving 85% of measurements error within 0.50 D from clinical standards (Salmeron-Campillo et al., 2025). Similarly, Luo (Luo et al., 2022) achieved strong spherical equivalent correlation (R = 0.91, LOAs = 0.17 ± 1.63). However, both methods lacked astigmatism measurement capabilities. In contrast, our study creatively used the astigmatic dial to acquire cylinder (C) diopter, addressing the gap in home-based vision screening.

A comprehensive assessment of the characteristics and performance metrics of this work as compared to other references is presented in Table 1.

**TABLE 1:** Comparison of the specifications between this study and other references.

Methods	Instruments	Ages	n	LOAs (D)	Time (mins)	Application scenario	VA/RE
Auto-refractor	BV-1000 (Dave and Fukuma, 2004)	38.6 ± 14.2 (16, 64)	100	0.05 ± 0.69	9.75 ± 0.18	Clinic	RE
WA/AO	VAO (Hervella et al., 2019)	34.6 ± 13.7 (21, 72)	76	0.00 ± 0.67	--	Clinic	RE
Phoropter	Easee (Wisse et al., 2019)	25.4 ± 4.7 (18, 40)	104	0.13 ± 1.22	22 ± 10	Home	RE
	EyeNetra (Tousignant et al., 2020)	(18, 35)	36	0.53 ± 1.40	--	Home	RE
Smartphone	EyeHandBook (Tofigh et al., 2015)	(18, 89)	100	0.09 ± 0.17*	--	Home	VA
	V@home (Han et al., 2019)	20.5 (13, 26)	50	−0.01 ± 0.24*	--	Home	VA
	Peek Acuity (Bastawrous et al., 2015)	∼65 (55, 97)	272	0.07 ± 0.41*	1.28 (1.18, 1.40)	Home	VA
	McAndrew (O'Neill and McAndrew, 2016)	54.5 ± 15 (24, 88)	120	ICC = 0.92*	--	Home	VA
	Salmerón-Campillo (Salmeron-Campillo et al., 2024)	21 ± 3	144	0.08 ± 0.67	--	Home	VA, RE
	Salmerón-Campillo (Salmeron-Campillo et al., 2025)	22.5 ± 3.4 (18, 43)	675	0.17 ± 0.84	--	Home	RE
	Luo (Luo et al., 2022)	22 ± 8.5 (6, 62)	201	0.17 ± 1.63	--	--	RE
	This work	21.36 ± 7.92 (7, 40)	460	0.10 ± 0.89	∼5	Home	RE

WA: Wavefront aberration; AO: Adaptive optics; VA: Visual acuity; RE: Refractive error. * LOAs were calculated using the difference values from LogMAR.

Traditional refractive screening models demand considerable time, labor, equipment, and administrative resources. In contrast, a forward-looking approach entails being precise, cost-effective, user-friendly, and capable of adapting to evolving myopia prevention needs. In response to this challenge, this paper introduces a smartphone-based subjective refraction method. Unlike traditional optometry models that necessitate expensive equipment and professional training, RE measurement using this method is straightforward. Users follow a step-by-step process, moving their smartphones to obtain their RE. The benefit of this method resides in its capability to assess changes in RE at any time and place, hence improving the efficiency of vision evaluation.

## 2 Methods

### 2.1 Subjects

This study recruited a cohort of 230 subjects, comprising 94 males and 136 females between 7 and 40 years old. Subjects were recruited from Tianjin Medical University Eye Hospital (Tianjin, China). The experiments were conducted at the optometry center. The study adhered to the principles set forth in the Declaration of Helsinki, with all subjects providing written informed consent. Ethical approval for the study was granted by the regional ethics committee. Inclusion criteria for participation in the study included individuals aged 6–40 years, S ≥ −6.00 D, C ≥ −6.00 D, and the absence of any other ocular pathologies. Eligible subjects (aged 6–40 years) were recruited. Exclusion criteria comprised: (1) ocular diseases other than refractive errors; (2) orthokeratology or soft contact lens use within 1 month preceding the study; (3) suspected unstable accommodation, defined as a spherical equivalent discrepancy of more than 0.50 D between objective autorefraction and final subjective refraction; and (4) inconsistent cooperation or unreliable subjective acuity responses.

### 2.2 Clinical measurements

The gold-standard clinical methodology for subjective refraction performed by experienced optometrists using a phoropter utilized a uniform and high-contrast ‘E’ chart with color temperature 4000–6000K, featuring black optotypes on a white background with a contrast ratio of approximately 1:100, which is 3 m far away subjects. To control pupil size and minimize accommodative fluctuations, the clinic clinical measurements is under standardized room illumination of 500 lux ensuring consistent and reliable results that served as the reference for validating the smartphone-based method.

### 2.3 Smartphone application

The innovative smartphone application (App) used artificial intelligence to recognize faces and speech. The App is compatible with a diverse range of smartphone brands, provided they run on iOS 8.0 (released in 2014) or Android 4.0 (released in 2011) or higher. The application managed inter-device luminance variability by utilizing the smartphone’s built-in ambient light sensor to dynamically adjust screen brightness (200–800 cd/m^2^), and minimized potential glare stimulation caused by significant brightness differences between environment and screen. It uses the phone’s built-in light sensor to check the surrounding light level, making sure it stays within a suitable range of 200–1,000 lux. The application can operate within a range of 200–1,000 lux. However, to ensure measurement accuracy by minimizing fluctuations in pupil diameter and accommodation. The recommended illumination is 500–600 lux to align with standard clinical practice.

The process of measuring the iris diameter (ID) in digital images, where ID refers to the pixel size in a digital image rather than the actual size in the real world, involves several steps to enhance image quality and measurement accuracy. Firstly, color facial images are transformed into grayscale to reduce image complexity and simplify analysis. Subsequently, histogram equalization is applied to enhance contrast, and make the iris and other features more distinguishable. The enhanced image was then subjected to segmentation, a crucial image recognition process where the iris region is isolated from the facial images. The algorithm evaluates the ID using circular fitting to ensure accuracy. Pupillary distance (PD) was then calculated by applying the pixel-to-millimeter ratio, derived from the measured iris diameter and its known average physiological size (∼12 mm) (Alotaibi et al., 2025), to the inter-pupillary distance in pixels. Diopter adjustments for S and C adhere to clinical standards (0.25 D increments), with raw calculations rounded to the nearest 0.25 D. Astigmatism axis (*α*) is measured in 10° intervals to balance precision and usability. It pays more attention that the subject should hold the smartphone as vertically as possible. According to the international standard of subjective refraction (Jacobs et al., 2023), subjects should vertically look at ‘E’ targets on screen. When iris images were captured as ellipse shape with smartphone tilting, the Hough-circle algorithm was implanted to fit ID, which will induce measurement error (SV, 2018; El-Sayed and Abdel-Latif, 2022; Zheng et al., 2022).

All subjective refractive tests are performed by experienced optometrists, and subjects are blinded to the results of all trials. Building on the relationship between the ID and the eye-to-smartphone screen distances (ESD), the prescription of RE and astigmatism is calculated by the ESD (Liu CH et al., 2012).

### 2.4 Procedure

The entire measurement setup and procedure were as follows: Prior to formal testing, all subjects received standardized training from an experienced optometrist. Each subject was instructed to stand during the test, holding the smartphone with both hands at eye level and keeping the screen as vertical as possible. No tripod was used. The optometrist verbally instructed participants to start from a distance where the ‘E' optotype was blurred and then “Gradually move the screen closer until you can clearly distinguish the orientation of the ‘E' optotype.” Subjects verbally reported the orientation of the ‘E' optotype while moving the device and paused for about 5 s at each position. During this pause, the system automatically scaled the optotype size based on real-time ESD data to maintain a 5-arcminute visual angle, after which the subject identified the orientation. This distance is defined as the far distance without accommodation.

**FIGURE 1:**
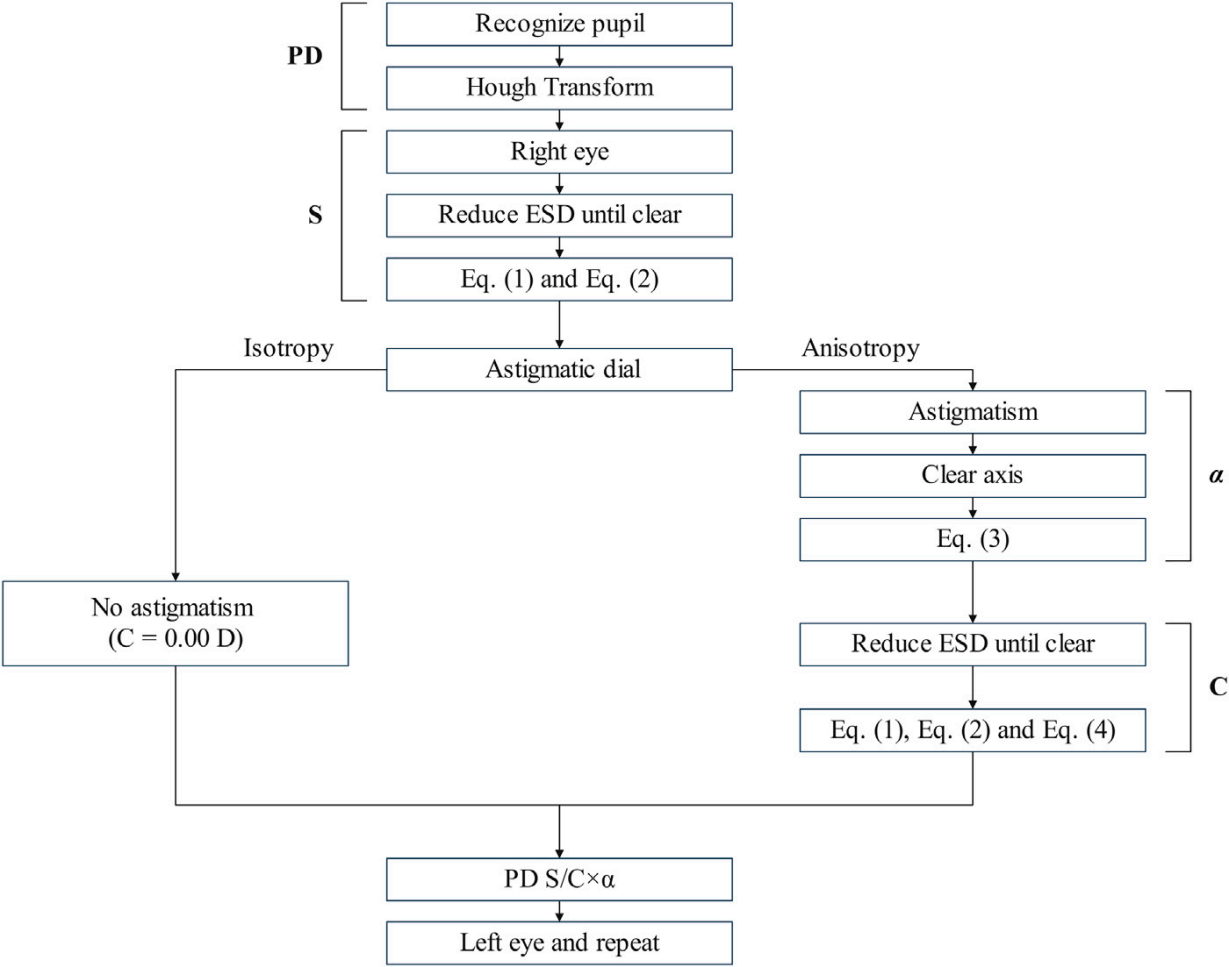
The flowchart of the subjective refraction based on the innovative refractive screening platform of smartphones.

Figure 1 shows the flowchart of the steps involved in the innovative refraction using smartphones. Initially, the system identified the pupil and measured PD. Subsequently, with the left eye covered to ensure monocular measurement, the following procedure was performed for the right eye. Initially, on-screen instructions guided the subject to hold the smartphone at a distance far enough with blurred the ‘E' optotypes for subject.

**FIGURE 2:**
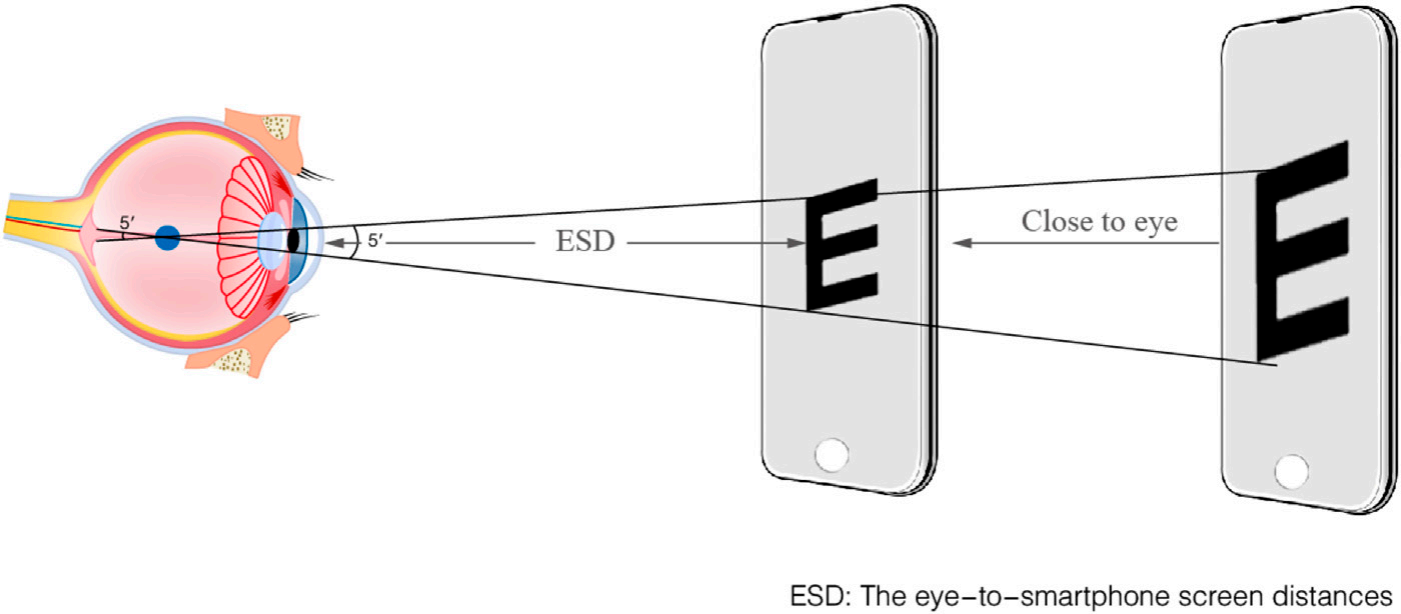
The schematic diagram of measuring the ESD.

The ‘E’ optotype is divided into five equal parts, with each segment forming a 1′ visual angle with the node point, which is the most consistent with the human eye’s resolution rule. ‘E’ optotypes always keep 5′ visual angle with the function of ESD and were displayed a sequence of 7‘E' optotypes with a random orientation. The detailed process of ESD is shown in Figure 2. Subjects verbally identified the opening direction for seven horizontally aligned optotypes per trial. A recognition threshold of ≥4 correct responses (≥4/7) validated 20/20 acuity equivalence. Spherical refractive error was derived from the ESD at threshold using Equation 1. Astigmatism-induced blur may transiently affect ‘E’ optotype clarity. The ≥4/7 recognition threshold ensures reliable S approximation even in the presence of uncorrected cylindrical error. The alignment with gold-standard refraction outcomes was assessed through a clinical validation procedure, as cylindrical correction is typically refined after spherical determination in standard workflows. The S diopter can be calculated using Equation 1 and Equation 2,

$$S=-\frac{1}{ESD}$$ (1)

$$ESD\left(m\right)=\frac{K}{ID}$$ (2)

where *K* represents the fitting coefficient obtained through the curve fitting between the ESD and the ID, ensuring the accuracy of the calculations. The coefficient *K* is determined based on the pixel specifications of the smartphone’s camera and its specific model, due to the variability in camera pixel specifications across different smartphones used by individuals. The coefficient *K* = 27.39 ± 2.07 is used to calibrate the PD pixel to real ESD distance by iPhone X in this study.

Following the measurement of ESD, subjects are instructed to switch to the astigmatism dial, as depicted in Supplementary Figure S1 The astigmatism dial is a semicircle marked with lines at 10° intervals. To make axis selection more intuitive for users without a clinical background, these lines are color-coded, providing a simple visual cue. This design allows users to perform an approximate estimation of astigmatic axis without the need for specialized clinical equipment.

If uniform line thickness is perceived in all directions by observers, the “spherical” prescription can be obtained directly, signifying the absence of astigmatism (C = 0.00 D). In contrast, if line thickness is uneven (“anisotropic”), it indicates the presence of astigmatism. According to the rule of thirty (Liu CH et al., 2012), the sharpest direction (*X*) can be ascertained based on the observer’s verbal indications, specifying the color or number on the astigmatism dial that appears most distinct. Subsequently, astigmatism axis (*α*) is calculated by Equation 3,

$$\alpha =\left\{\begin{array}{l} 90{}^{\circ}-\left(X-1\right)\times 10{}^{\circ},X\in \left[1,10\right]\\ 270{}^{\circ}-\left(X-1\right)\times 10{}^{\circ},X\in \left[11,18\right] \end{array}\right.$$ (3)

where *X* represents the sharpest line (direction) as indicated by the observer. The astigmatism dial was then progressively moved towards the observer by themselves, until the lines at the orthogonal direction became the sharpest. At this position, the ESD is conjugated with the anterior focal line on the retina. The calculation of the C diopter is achieved using Equation 1 and Equation 4,

$$C=S_{2}-S_{1}$$ (4)

where S_1_ and S_2_ are the refraction errors for the posterior and anterior focal lines. The process commences by defining the ESD_1_ as the maximum distance at which the optotype remains clearly visible, which is conjugate to the posterior focal plane of the retina. Then, the observer manually moves the astigmatism dial closer to themselves, guided by the direction (*X*) that appears the sharpest. The procedure is continued until lines orthogonal to the initial direction emerge as the most distinct. At this juncture, the distance (ESD_2_) indicates the position to calculate the refraction error associated with the anterior focal conjugate. Herein, S_1_ and S_2_ denote the refraction errors conjugate to the posterior and anterior focal planes, respectively. After ascertaining the right eye prescription through this approach, the procedure is replicated for the left eye, ensuring monocular measurement by obscuring the right eye. The smartphone-based measurement aligns with subjective refraction principles, requiring subjects to iteratively adjust the screen position until achieving optimal clarity of the ‘E’ optotype and astigmatic dial lines by subjective perception.

### 2.5 Statistical analysis

All statistical analyses were performed using SPSS version 27 (IBM, United States) and OriginPro 2024 (OriginLab, United States). A *p*-value of less than 0.05 was considered statistically significant. To evaluate the agreement between the smartphone and the clinical method, Bland-Altman analysis was employed. The normality distribution of the differences for all parameters was first assessed using the Shapiro-Wilk test. For each parameter, the difference was calculated as (Smartphone Value - Clinical Value). The following metrics were determined: Mean Difference (MD) to quantify systematic bias, the 95% Confidence Interval (CI) of the MD, and the 95% LOAs, calculated as MD±1.96 × SD of the differences. A paired *t*-test was also used to determine if the MD was statistically significantly different from zero.

For a more comprehensive analysis of astigmatism, the clinical refraction data (S, C, *α*) were converted into power vector components (M, J0, J45) (Thibos et al., 1997) using standard formulas (Equations 5–7), with C taken as a negative value:

$$\text{SER}=S+\frac{C}{2}$$ (5)

$$J0=-\frac{C}{2}\cos2\alpha$$ (6)

$$J45=-\frac{C}{2}\sin2\alpha$$ (7)

Agreement for these components was then assessed using the same Bland-Altman methodology.

For subgroup comparisons, such as between youth and adult groups, an independent samples *t*-test was used. To assess interocular consistency, a paired *t*-test was used to compare the measurements between the right and left eyes for both the clinical and smartphone methods.

To evaluate the clinical utility of the smartphone method as a screening tool, its diagnostic performance for detecting specific ranges of myopia was assessed using Receiver Operating Characteristic (ROC) curve analysis. In this context, the smartphone-measured SER was treated as the diagnostic test variable. Performance was quantified by the Area Under the Curve (AUC), which represents the probability of correctly ranking a positive case higher than a negative case.

## 3 Results and discussion

The subjects were categorized into two groups: teenagers (≤18 years old) and adults (>18 years old). There are no adverse events or complications recorded during the study. Table 2 provides an overview of the baseline characteristics between the two groups.

**TABLE 2:** The baseline clinical characteristics of the study population.

Characteristic	Mean (SD)		
	Teenagers (n = 101)	Adults (n = 129)	Total (n = 230)
Age, y	14.65 (3.03) (7, 18)	26.05 (6.67) (19, 40)	21.04 (7.76) (7, 40)
Sex, No. (%)			
Male	45 (44.55)	49 (37.98)	94 (40.87)
Female	56 (55.45)	80 (62.02)	136 (59.13)
PD (mm)	60.85 (3.76) (47.00, 70.00)	62.75 (2.92) (54.00, 71.00)	61.92 (3.44) (47.00, 71.00)
S (D)	−1.68 (1.64) (-6.00, 0.50)	−2.56 (1.65) (-6.25, 0.25)	−2.17 (1.70) (-6.25, 0.50)
C (D)	−0.39 (0.51) (-2.00, 0.50)	−0.44 (0.50) (-2.50, 0.00)	−0.41 (0.51) (-2.50, 0.50)
J0 (D)	−0.03 (0.18) (-0.87, 0.76)	−0.04 (0.22) (-1.24, 0.75)	−0.03 (0.20) (-1.24, 0.76)
J45 (D)	0.04 (0.26) (-0.72, 0.84)	0.03 (0.25) (-0.60, 1.08)	0.03 (0.26) (-0.72, 1.08)
SER (D)	−1.87 (1.74) (-6.25, 0.50)	−2.77 (1.70) (-6.62, 0.00)	−2.38 (1.77) (-6.62, 0.50)
Myopia^a^ parents, No. (%)			
0	58 (57.43)	102 (79.07)	160 (69.57)
1 or 2	43 (42.57)	27 (20.93)	70 (30.43)
Astigmatism parents, No. (%)			
0	73 (72.28)	100 (77.52)	173 (75.22)
1 or 2	28 (27.72)	29 (22.48)	57 (24.78)
Wearing Glasses (y)	1.95 (2.45) (0, 10)	5.79 (5.43) (0, 25)	4.11 (4.77) (0, 25)

SD: standard deviation.

(min, max): The interval range between minimum and maximum values.

^a^ The threshold for myopia is defined as S ≤ −0.50 D.

This study included a total of 460 eyes from 230 subjects, comprising 94 males and 136 females. The mean age of the subjects was 21.04 ± 7.76 years, with an age range spanning from 7 to 40 years. The mean S diopter was −2.17 ± 1.70 D, ranging from −6.25 to +0.50 D. The mean C diopter was −0.41 ± 0.51 D, within a range of −2.50 to 0.00 D. The mean SER was −2.38 ± 1.77 D, covering a range from −6.62 to +0.50 D. The proportion of teenagers and adults without myopic parents were 57.43% and 79.07%, respectively. It was noteworthy that the myopia rate among parents in the teenage group remarkably exceeded that in the adult group, which indicated a concerning trend of worsening myopia among younger generations. The percentage of subjects’ parents without astigmatism stood at 72.28% and 77.52% for teenage and adult groups, respectively. Notably, there was no significant shift in the prevalence of astigmatism across age groups, as measured by traditional subjective refraction. On average, subjects reported wearing glasses for a period of 4.11 ± 4.77 years, ranging from 0 to 25 years.

**FIGURE 3:**
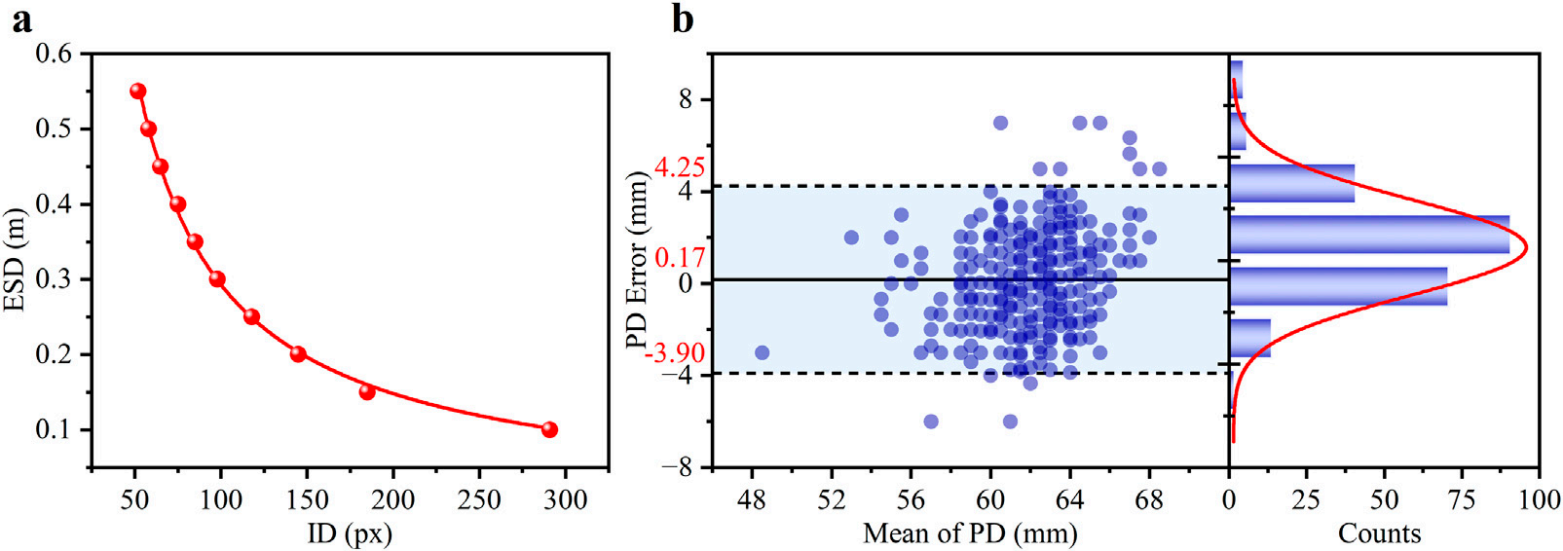
**(a)** The experimental scatter data is the function of the ID and the ESD and the corresponding fitted curve (red line). **(b)** Bland-Altman analysis of the agreement in PD measurements between the smartphone and clinical methods. The left panel shows the Bland-Altman plot with the mean difference (solid line) and 95% limits of agreement (dashed lines). The right panel shows the histogram of the differences, with a Gaussian fit curve (red line).

The relationship between the ESD and the ID was illustrated in Figure 3a, clearly showing an inverse relationship. The coefficient of determination (R^2^) for the fitting curve exceeded 0.99, indicating an exceptional fit between the regression line and the experimental values. In children population, the ID was 10.54 ± 0.43 mm for 6–10 years old, and 10.73 ± 0.36 mm for 11–15 years old. (Challa et al., 2024). When the children grew, the change of ID was minimal with measurement error <4% which furtherly supporting smartphone method to obtain refractive error.

It was essential to ensure precise distance inversion to evaluate the feasibility of utilizing ID to determine RE. PD errors of the subjects, calculated by comparing the pupillometry method (considered the gold standard in clinical settings), were used to verify the accuracy of the smartphone-based ID measurement.

The density distribution of the PD error for the 460 eyes was presented in the left section of Figure 3b, where the scatter data was primarily concentrated in the range of ± 4 mm. The statistical histogram was fitted with a Gaussian distribution, depicted as a red curve. The difference plot demonstrated a strong level of agreement between the novel smartphone-based approach and the pupillometer measurements. A Bland-Altman analysis revealed a small mean bias of 0.17 mm with 95% LOAs of ±4.08 mm (mean ± 1.96 SD). These results indicate that the smartphone-based method is highly capable of providing accurate distance measurements.

**FIGURE 4:**
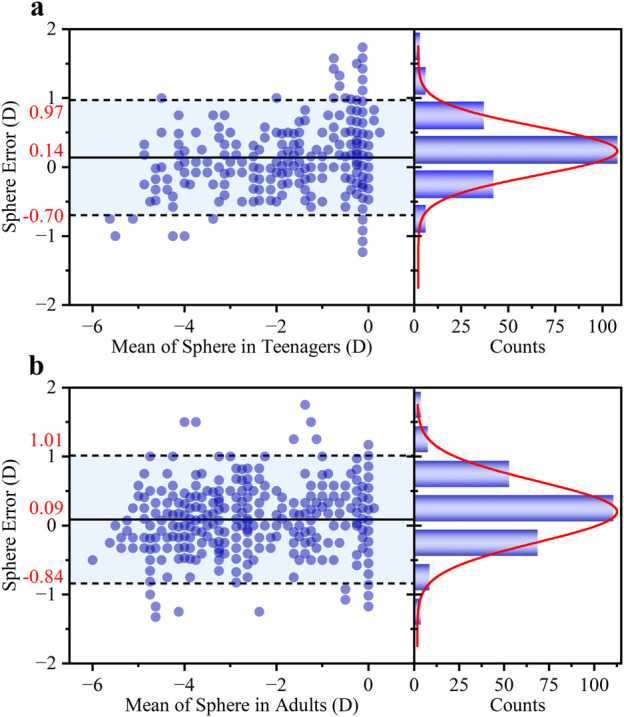
Bland-Altman analysis of the agreement in S diopter measurements between the proposed method and traditional refraction for **(a)** teenagers and **(b)** adults. For each group, the left panel shows the Bland-Altman plot with the mean difference and 95% LOAs, while the right panel shows the histogram of the differences with a corresponding Gaussian fit curve (red line).

As shown in Figure 4, the agreement between the S diopter measurement and clinical subjective refraction was high for both groups. For the teenage group (a), the Bland-Altman analysis revealed a small mean bias of +0.14 D with 95% LOAs of ±0.83 D. Crucially, 86.14% of measurements fell within the clinically acceptable error margin of ±0.50 D. The adult group (b) demonstrated comparable performance, with a mean bias of +0.09 D, 95% LOAs of ±0.93 D, and 85.66% of errors within ±0.50 D.

The larger measurement error observed in the adult group may be attributed to two main factors. Firstly, the sample characteristics differed; the adult group predominantly consisted of individuals with mild to moderate myopia, whereas the younger group had a higher proportion of low myopia. Secondly, the relationship between ESD and S is inherently non-linear. As defined by calculation method (Equation 1), S is inversely proportional to ESD. This means that the ESD shift will induce the calculated diopter error for high myopia (smaller ESD).

**FIGURE 5:**
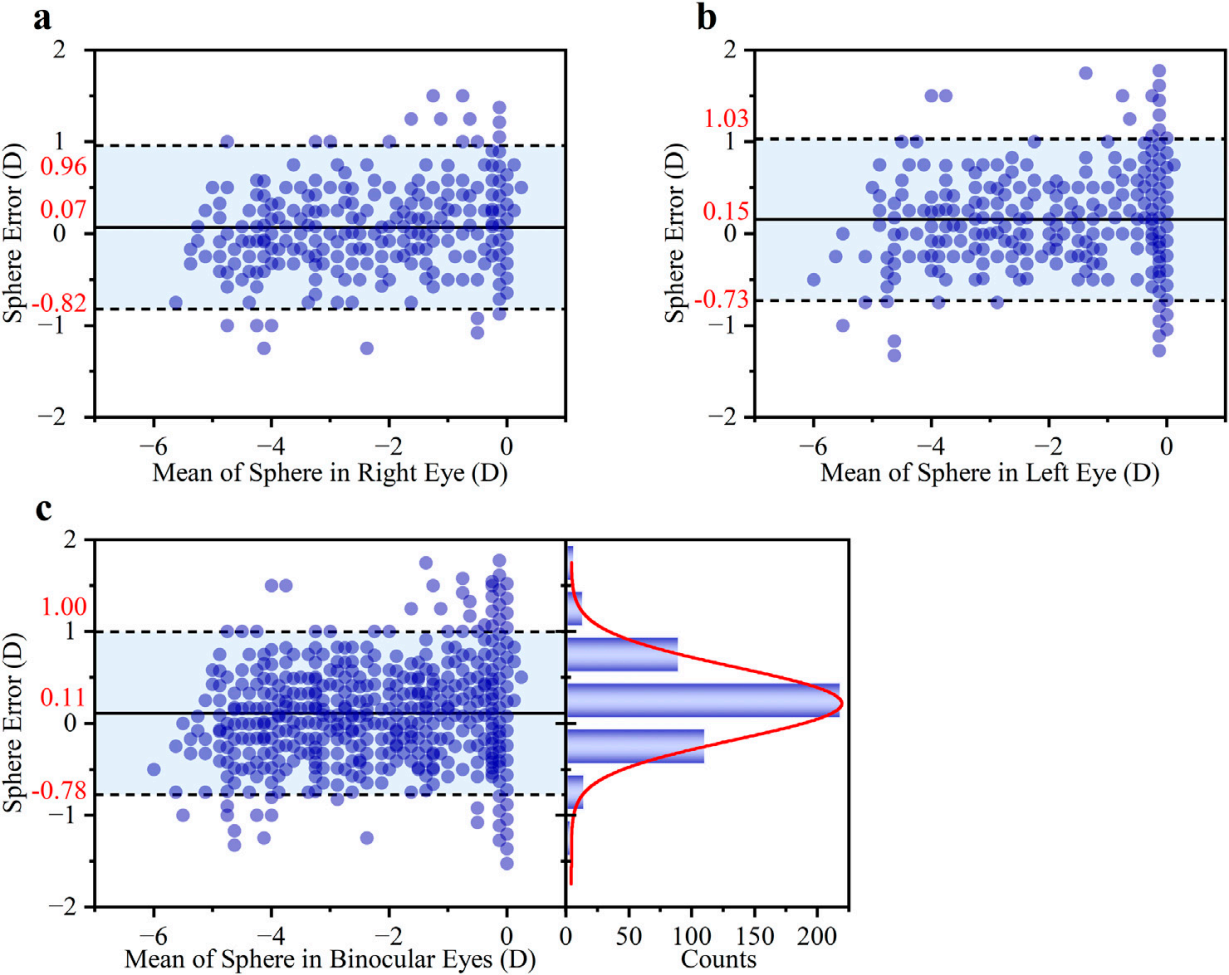
Bland-Altman analysis of the agreement in S diopter measurements. The analysis is presented for **(a)** the right eye, **(b)** the left eye, and **(c)** both eyes. Each subgroup includes a Bland-Altman plot (left) showing the mean difference and 95% LOAs, and a histogram of the differences (right) with an overlaid Gaussian curve (red line).

The performance of the smartphone method in measuring S diopter was highly consistent between the right and left eyes, as shown in Figure 5 Bland-Altman analysis. Specifically, the mean difference (±95% LOAs) was 0.07 ± 0.89 D for the right eye and 0.15 ± 0.88 D for the left eye. The close similarity in both the mean differences, which indicate minimal systematic bias, and the width of the LOAs, suggests that the method’s accuracy and precision are independent on binocular. The overall mean difference was 0.11 ± 0.89 D for both eyes in Figure 5c. Furthermore, a high degree of clinical agreement was observed, with 85.87% of the measurement differences falling within the clinically acceptable range of ±0.50 D.

**FIGURE 6:**
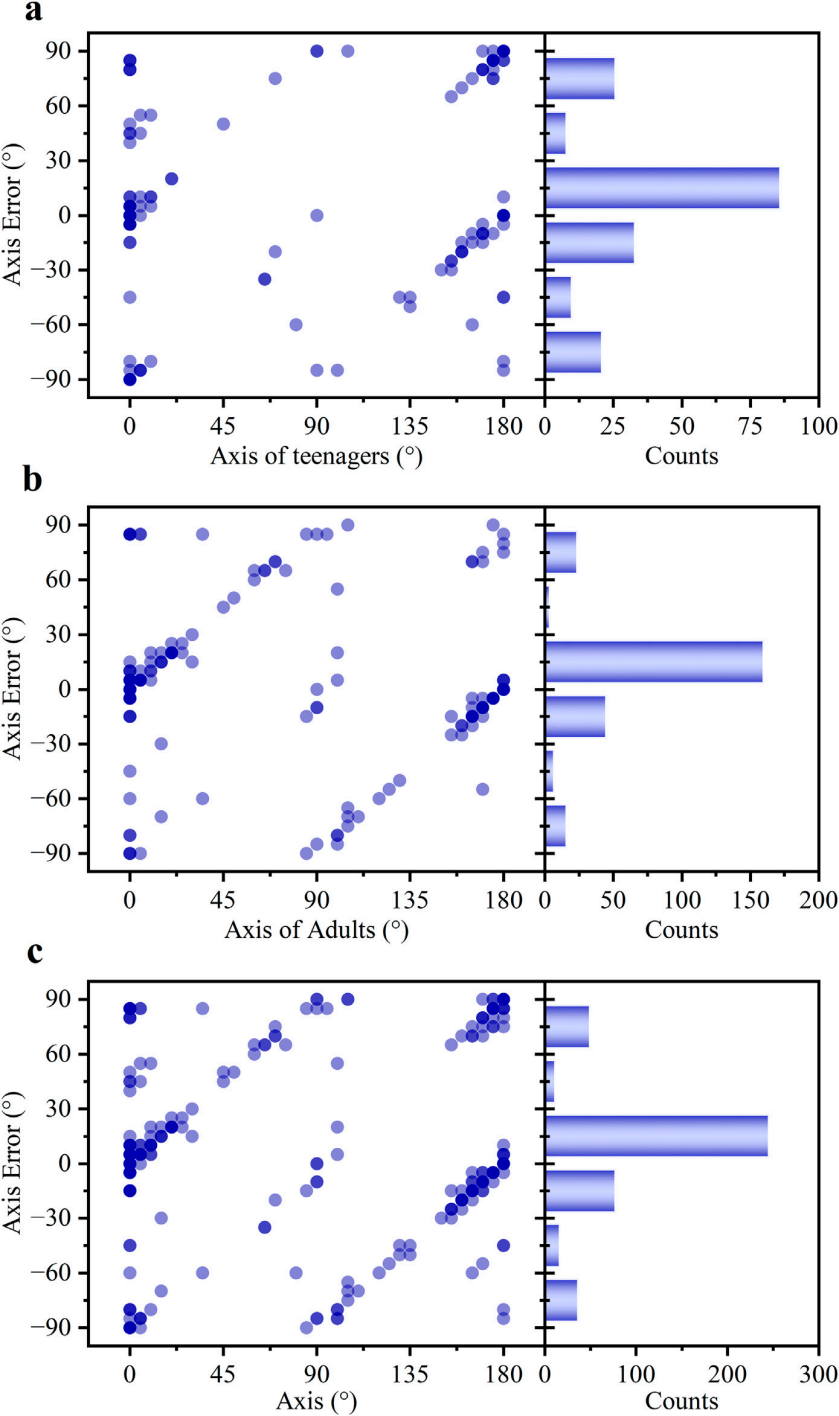
The statistical profiles of the *α* error for **(a)** teenagers, **(b)** adults, and **(c)** all subjects.

As shown in Figure 6, a noticeable difference in astigmatic axis (*α*) measurement accuracy was observed between the two age groups. The average deviation in the teenage group was 7.95°, significantly higher than the 2.19° observed in the adult group. Consequently, only 53.96% of axis measurements in the teenage group fell within the clinically accepted ±15° range, compared to 72.48% in the adult group. Overall, the pooled data showed an average axis deviation of 4.72°, with 64.35% of measurements within the ±15° range (Figure 6c).

For a more rigorous assessment of astigmatic agreement, power vector analysis was conducted (Supplementary Figure S2). The Bland-Altman plot for the J0 component (Supplementary Figure S2A) demonstrated excellent agreement, with a clinically negligible mean difference of +0.02 D. The 95% LOAs were narrow at [-0.50 D, +0.54 D]. Similarly, the analysis for the J45 component (Supplementary Figure S2B) revealed a minimal mean difference of +0.03 D and narrow 95% LOAs of [-0.59 D, +0.65 D], confirming high precision with no significant bias towards oblique astigmatism.

**FIGURE 7:**
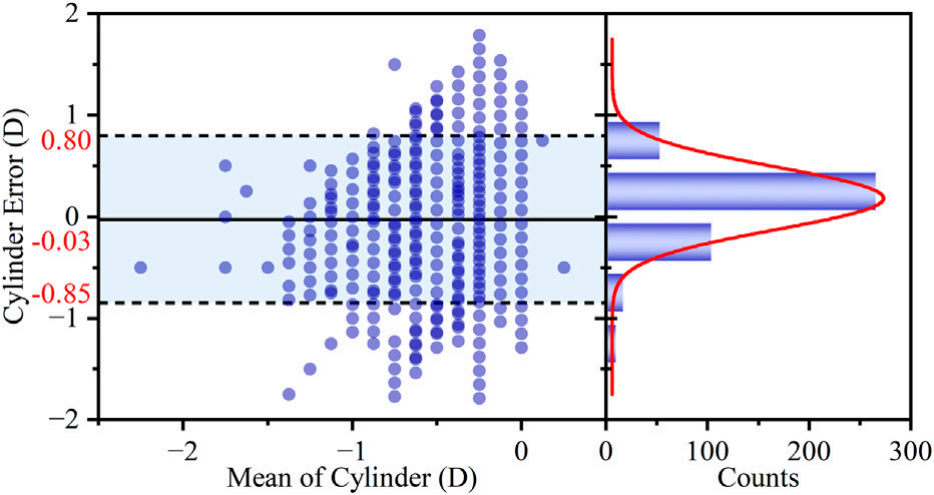
Bland-Altman analysis of the agreement in C diopter measurements between the two methods. The left panel shows the Bland-Altman plot with the mean difference (bias) and 95% LOAs. The right panel displays the histogram of the differences, with an overlaid Gaussian curve (red line).

The agreement in C diopter measurements was evaluated using a Bland-Altman plot (Figure 7). The analysis demonstrated excellent agreement between the smartphone and clinic method. The mean difference was merely −0.03 D, which is clinically negligible and indicates a lack of systematic bias in the measurements. Furthermore, the LOAs were [-0.85 D to 0.80 D], indicating good precision for a screening application. The clinical utility was further supported by the high proportion of measurements showing close agreement, with 89.57% of the differences falling within the narrow range of ±0.50 D.

**FIGURE 8:**
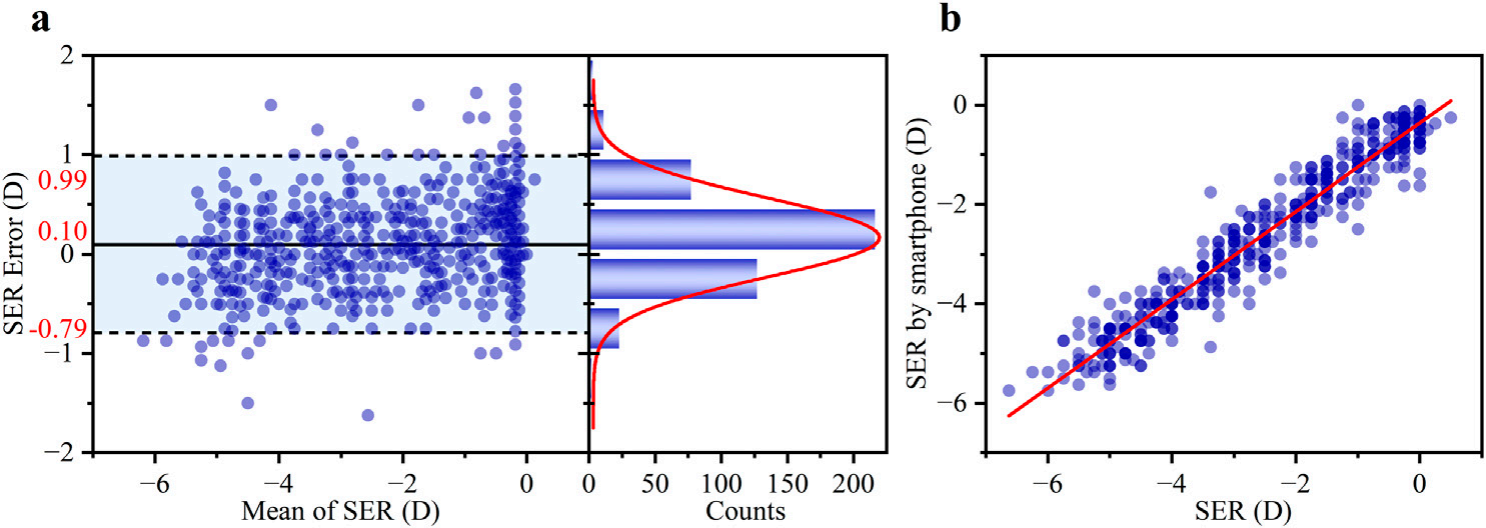
**(a)** Bland-Altman analysis showing the mean difference and 95% LOAs (left panel), and the corresponding histogram of differences with a Gaussian fit curve (right panel, red line). **(b)** Comparison of the SER error between the smartphone-based results and the subjective refraction standard. Blue scatter points represent individual measurements from 460 eyes. The red line shows the least-squares linear regression fit (R^2^ = 0.94).

The performance of the smartphone-based SER measurement was evaluated using both agreement and correlation analyses. A Bland-Altman analysis (Figure 8a) was conducted to assess agreement with the clinical subjective refraction. This revealed a small mean bias of +0.10 D, with 95% LOAs of ±0.89 D. Critically, 81.30% of the measurement errors fell within the clinically acceptable range of ±0.50 D, demonstrating strong consistency with the clinical approach. A linear regression analysis (Figure 8b) was performed to evaluate the correlation. The scatter plot in Figure 8b displays the experimental results. Overlapping data points are visualized using transparency, with areas of high density reflecting a strong concordance between the two methods. The smartphone measurements were highly correlated with the actual subjective refraction, with data points clustering tightly around the regression line (R^2^ = 0.94). The slope of the line was 0.89 ± 0.01, indicating a strong linear relationship close to the ideal 1:1 line. The standard deviation of the SER error was 0.45 D over the measured range of −6.625 D to +0.50 D.

Comparative analysis between teenage (n = 202) and adult (n = 258) groups revealed no statistically significant difference in SER error measurements (*p* > 0.05), with mean differences of 0.14 D and 0.06 D, and 95% LOAs of 0.14 ± 0.85 D and 0.06 ± 0.92 D for teenagers and adults, respectively, confirming consistent performance across age groups. The detailed results of the SER agreement for the teenage and adult subgroups are presented in Supplement Letter.

For paired comparisons, the mean difference, the 95% confidence interval (CI), and the 95% LOAs between the smartphone and clinical methods were calculated for PD, S, J0, J45, and SER. Detailed results are presented in Table 3. The mean difference in PD was 0.17 mm (95% CI, −0.10–0.44 mm), and this difference was not statistically significant. When comparing S values, the mean difference was 0.14 D (95% CI, 0.08 to 0.20 D) for teenagers and 0.09 D (95% CI, 0.03 to 0.15 D) for adults. Importantly, according to independent samples *t*-test analysis, the difference in performance between these two groups was not statistically significant (*p* = 0.236), suggesting the platform’s consistent applicability across the studied age ranges. For astigmatism, vector analysis using J0 and J45 components demonstrated excellent agreement. The mean difference was a mere 0.02 D for J0 and 0.03 D for J45. Excellent agreement was also found for SER, with a mean difference of 0.10 D (95% CI, 0.05 to 0.14 D). The 95% LOAs confirmed a high level of precision suitable for a screening device: SER ([-0.79, 0.99] D), J0 ([-0.50, 0.54] D), and J45 ([-0.59, 0.65] D). While the paired *t*-tests indicated that the differences for S, J45, and SER were statistically significant (*p* < 0.05), small *p*-values are has not a clinically meaningful with small MD±SD, which the negligible mean differences highly demonstrate agreement between both methods.

**TABLE 3:** Bland-Altman agreement analysis and paired *t*-test comparison between smartphone and clinical measurements.

Features	Group	n	Mean difference (MD)^a^	95% CI for MD	95% LOAs^b^	*p*-value^b^
PD (mm)	Overall	230	0.17	(-0.10, 0.44)	(-3.90, 4.25)	0.206
S (D)	Youths	202	0.14	(0.08, 0.20)	(-0.70, 0.97)	<0.05^c^
	Adults	258	0.09	(0.03, 0.15)	(-0.84, 1.01)	<0.05^c^
	Overall	460	0.11	(0.07, 0.15)	(-0.78, 1.00)	<0.05^c^
C (D)	Overall	460	−0.03	(-0.06,0.01)	(-0.85, 0.80)	0.148
J0 (D)	Overall	460	0.02	(-0.01, 0.04)	(-0.50, 0.54)	0.174
J45 (D)	Overall	460	0.03	(0.00, 0.06)	(-0.59, 0.65)	<0.05^c^
SER (D)	Overall	460	0.10	(0.05, 0.14)	(-0.79, 0.99)	<0.05^c^

^a^ Mean Difference (MD) calculated as (Smartphone Value - Clinical Value).

^b^ *p*-value derived from a paired *t*-test comparing two methods.

^c^ denotes statistical significance (*p* < 0.05).

**FIGURE 9:**
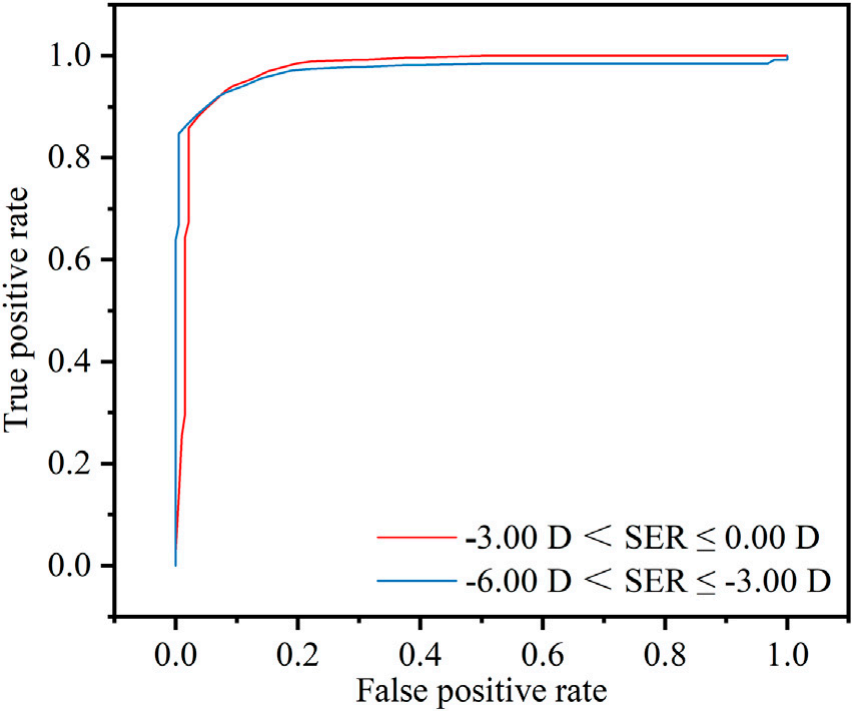
Threshold of −3.00 D < SER ≤ 0.00 D (red line) or −6.00 D < SER ≤ −3.00 D (blue line), diagnostic values and receiver operating characteristic (ROC) analysis of the refractive screening base on the smartphone.

The outcomes concerning the predictive probabilities of the SER were presented in Figure 9. For the range −3.00 D < SER ≤ 0.00 D (the red line), the detection model exhibited an AUC of 0.973, with an optimal cut-off value of −2.81 D, corresponding to a sensitivity of 93.3% and a specificity of 91.7%. For the range −6.00 D < SER ≤ −3.00 D (the blue line), the AUC was 0.986, also with an optimal cut-off value of −2.81 D, associated with a sensitivity of 93.3% and a specificity of 93.2%.

In the realm of primary eyecare, the integration of smartphone technology for refractive screening represents a significant advancement. While existing methods primarily focus on VA assessment using Snellen optotypes on smartphones, this method uses advanced image analysis algorithms to derive a series of essential parameters for refractive management, i.e., PD, S, C, and *α*.

In comparison to other smartphone-based approaches, the present method offers distinct advantages. Salmerón-Campillo et al. (Salmeron-Campillo et al., 2024; Salmeron-Campillo et al., 2025) leverage LCA which inherently dependents on specific screen color spectra and luminance, thus limiting its compatibility across diverse device types. Luo et al. (Luo et al., 2022) are unable to measure astigmatism, and the accuracy of SER significantly diminishes for astigmatism exceeding −1.75 D. In contrast, this platform calculates RE (S, C, and *α*) by deriving the far point from the ESD. This principle is independent of chromatic dispersion, is compatible with various screen types, and provides a more clinically comprehensive measurement by including astigmatism. Despite these methodological differences, this study results demonstrate clinical agreement with recent studies, as evidenced by SER LOAs of 0.10 ± 0.89 D.

There are some minimal limitations about this method. A limitation of the current study is the lack of a personalized calibration routine for individual anatomical variations in ID and ESD, which represents an opportunity for future development. However, evidence from the literature indicates that iris diameter typically measures 11.95 ± 0.39 mm (Alotaibi et al., 2025). The observed 3.3% deviation in personalized iris diameter estimation remains within an acceptable range of measurement error. The measurement accuracy may be affected by teenagers’ cognitive and cooperative ability. The perceptual task of judging the sharpest line on the astigmatism dial can be challenging for younger subjects. This study was conducted without cycloplegia, meaning accommodation maybe not fully control. This could lead to a myopic shift in the measurements, potentially causing an overestimation of myopia. These factors require continuous improvement of the interface guidance provided by smartphones to users. The current version does not include explicit calibration for differences between OLED and LCD screens, which may introduce minor systematic errors in ESD calculations due to variations in angular luminance dependence or spectral output. This application has not been validated under conditions of extremely low or high illumination, which may induce pupillary and accommodation response and have adversely effect on measurement accuracy.

Looking forward, this platform has the potential to democratize myopia screening. Its performance as a screening tool is exceptionally strong, confirmed by its excellent diagnostic accuracy for identifying clinically relevant myopia (AUC > 0.97). The smartphone-based approach will pave the way for widescale improvements in early myopia detection and prevention.

## 4 Conclusion

This study pioneers the validation of an innovative refractive screening method by smartphone. Through the implementation of sophisticated image analysis algorithms for iris recognition, the system can obtain the ESD, consequently enabling accurate calculation of the refractive error. This method demonstrates a remarkable level of concurrence with the gold-standard subjective refraction in clinical work. The LOAs for the S, the C, and the SER are 0.11 ± 0.89 D, −0.03 ± 0.82 D, and 0.10 ± 0.89 D, respectively. The average difference in *α* is 4.72°, with a substantial proportion (64.35%) of deviations falling within the ±15° range. Additionally, the innovative refractive screening displays impressive AUC values of 0.973 for the range of −3.00 D < SER ≤ 0.00 D and 0.986 for the range of −6.00 D < SER ≤ −3.00 D. The real-time and portable attributes of smartphones hold the potential to facilitate widespread, routine vision assessment, thereby assuming a pivotal role in myopia screening.

## Data Availability

The original contributions presented in the study are included in the article/Supplementary Material, further inquiries can be directed to the corresponding authors.
