# Supplementary material for: Innovative myopic screening platform based on smartphones
Source: Front Bioeng Biotechnol. 2025 Oct 20;13:1678800. doi: 10.3389/fbioe.2025.1678800 (PMC12580630; doi:10.3389/fbioe.2025.1678800)
Supplement: Supplementary file 1 [file DataSheet2.pdf]

1. Homepage

Homepage for first-time login & myopia warning deactivated

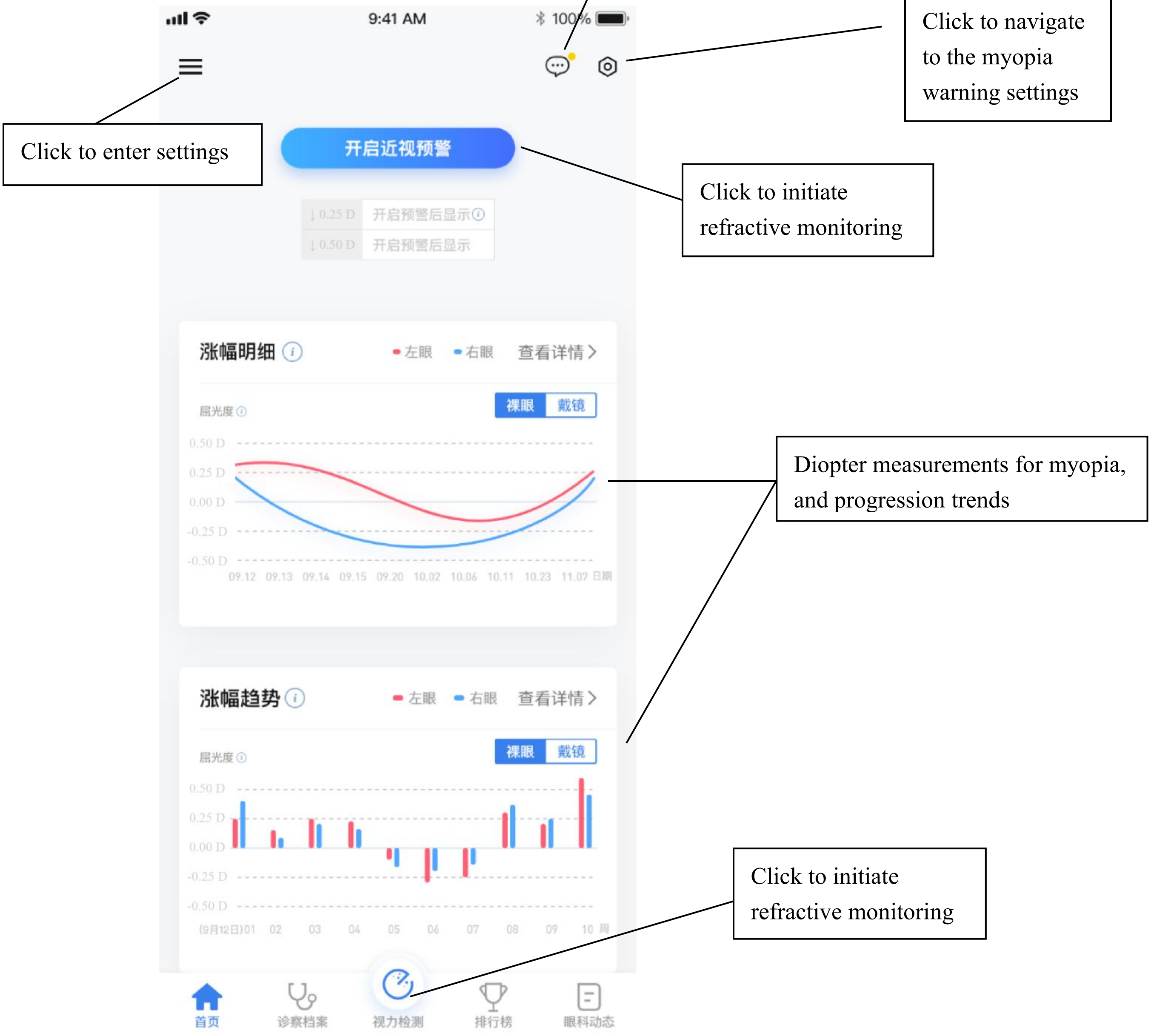

Figure A-1

Homepage with refractive detection activated

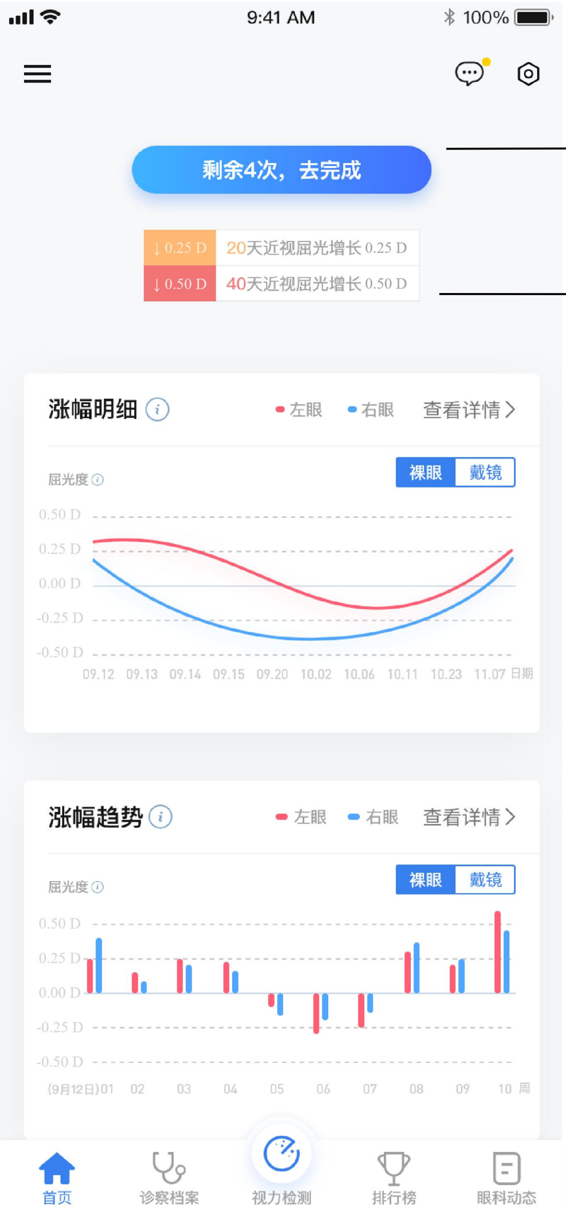

Upon completion of the refractive detection, the backend issues the corresponding data.

Calculate the myopia diopter change relative to the previous measurement date based on data delivered from the backend.

Figure A-2

2. Homepage - Myopia warning settings

Clicking the “Initiate refractive monitoring” button on the homepage navigates to Figure B-1 (requires the user to grant microphone and camera access permissions). Clicking the back arrow on any page will return to the Homepage (Figure A) with refractive detection deactivated.

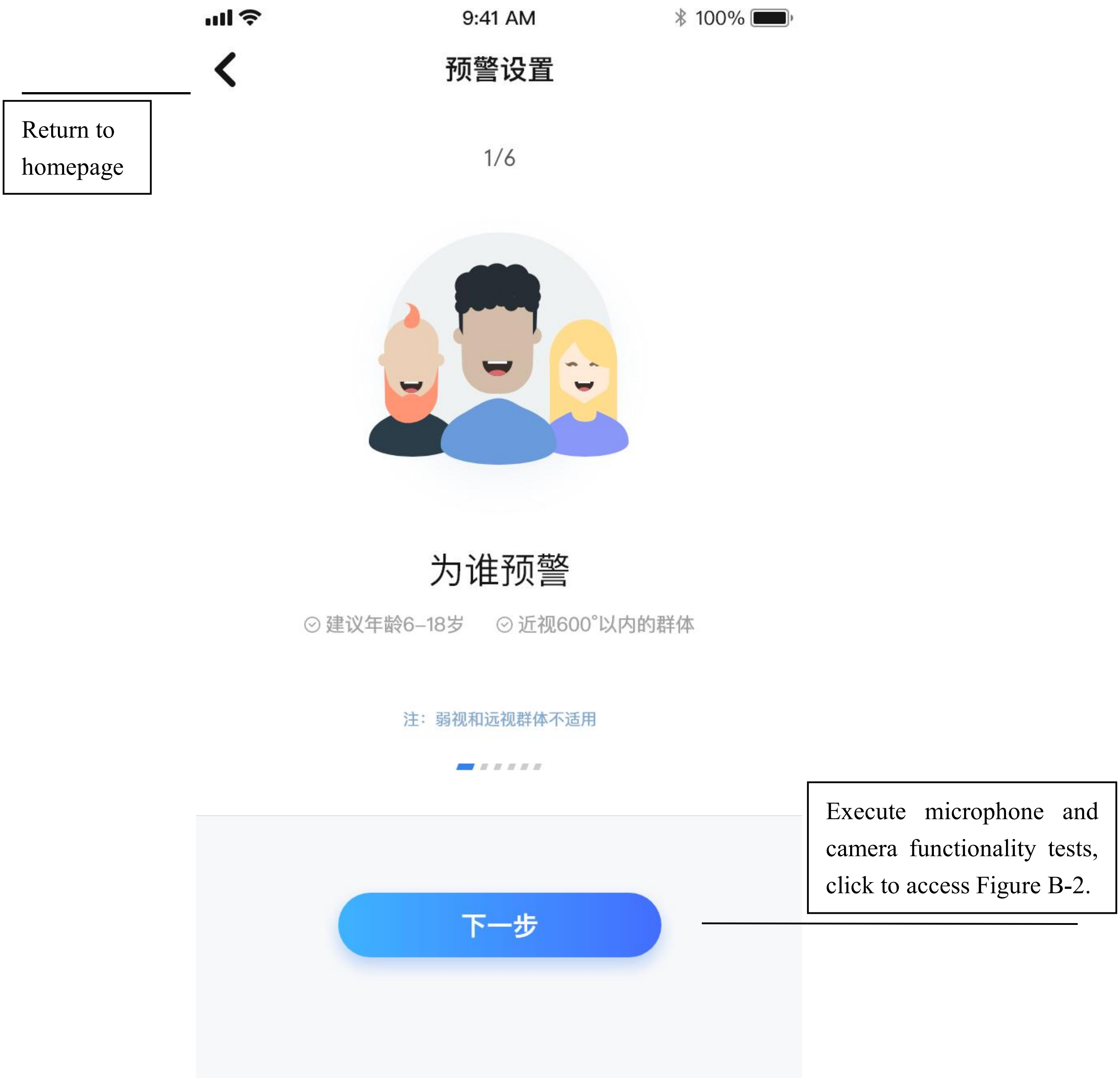

Figure B-1

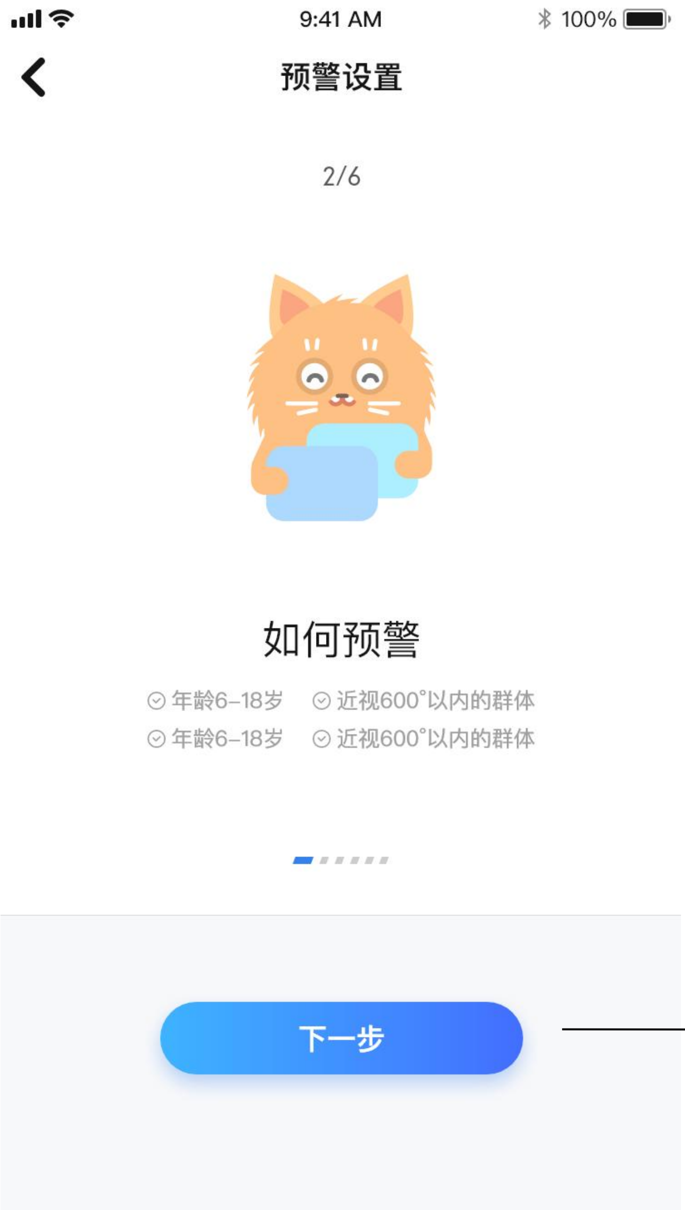

Step-by-step operational guide, click to access Figure B-3.

Figure B-2

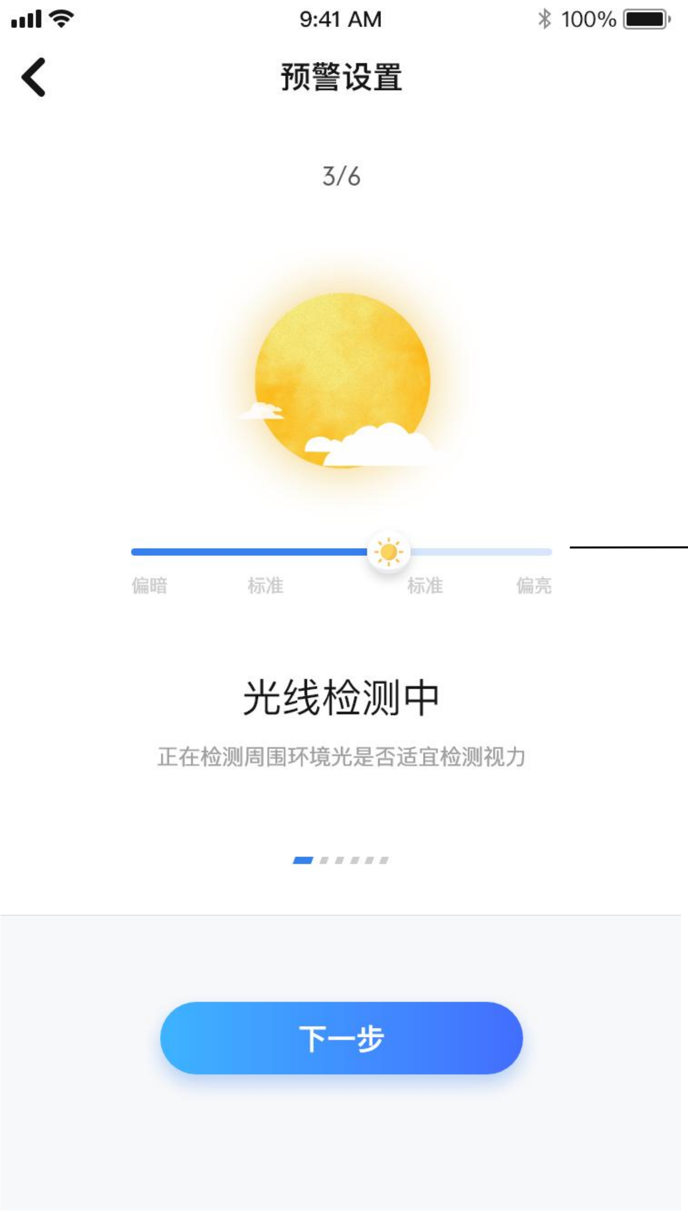

When the front camera's ambient light sensor is activated to initiate ambient light detection, the interface dynamically adjusts the lighting indicator's visual state: if the measured illuminance falls outside the predefined standard range (too dim or exceeding the upper threshold), the indicator area appears dimmed (grayed-out) or excessively highlighted, while the 'Next' button remains disabled (grayed-out); once the illuminance value stabilizes within the compliant range, the 'Next' button transitions to an enabled state (blue and clickable).

Figure B-3-1

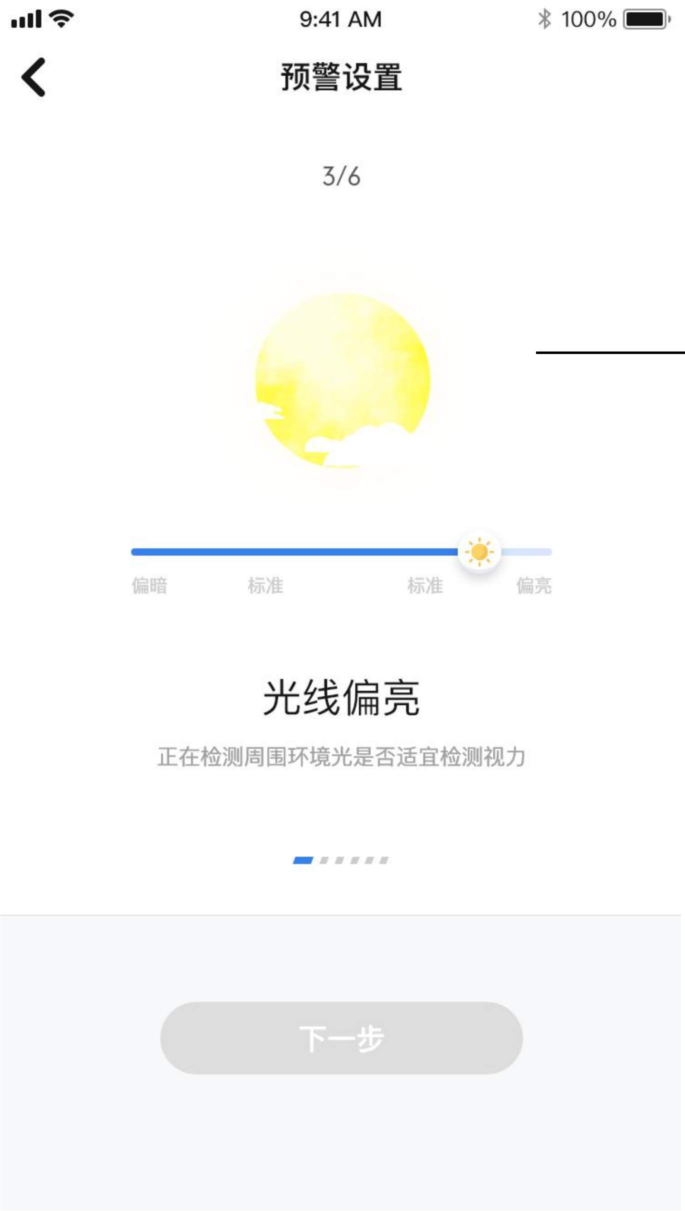

Taking the case where illuminance exceeds the upper threshold as an example, the sun icon's brightness level increases in the user interface.

Figure B-3-2

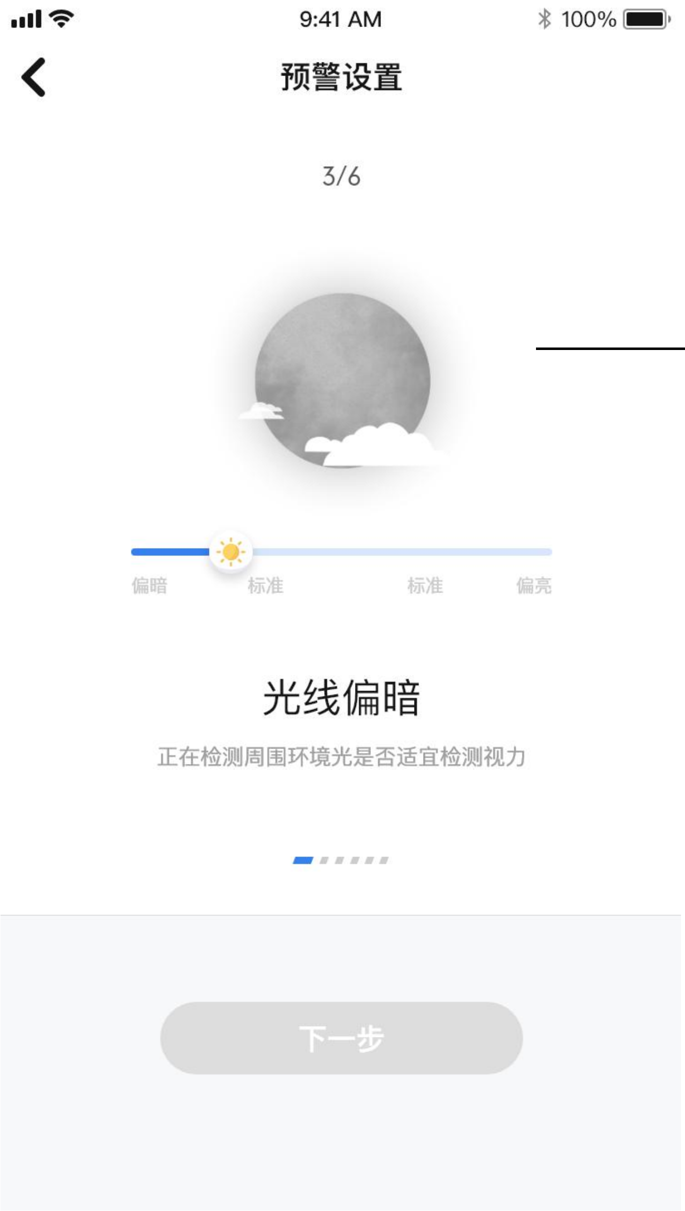

Taking the scenario of insufficient ambient illuminance as an example, the sun icon's visual brightness level is correspondingly reduced in the UI display

Figure B-3-3

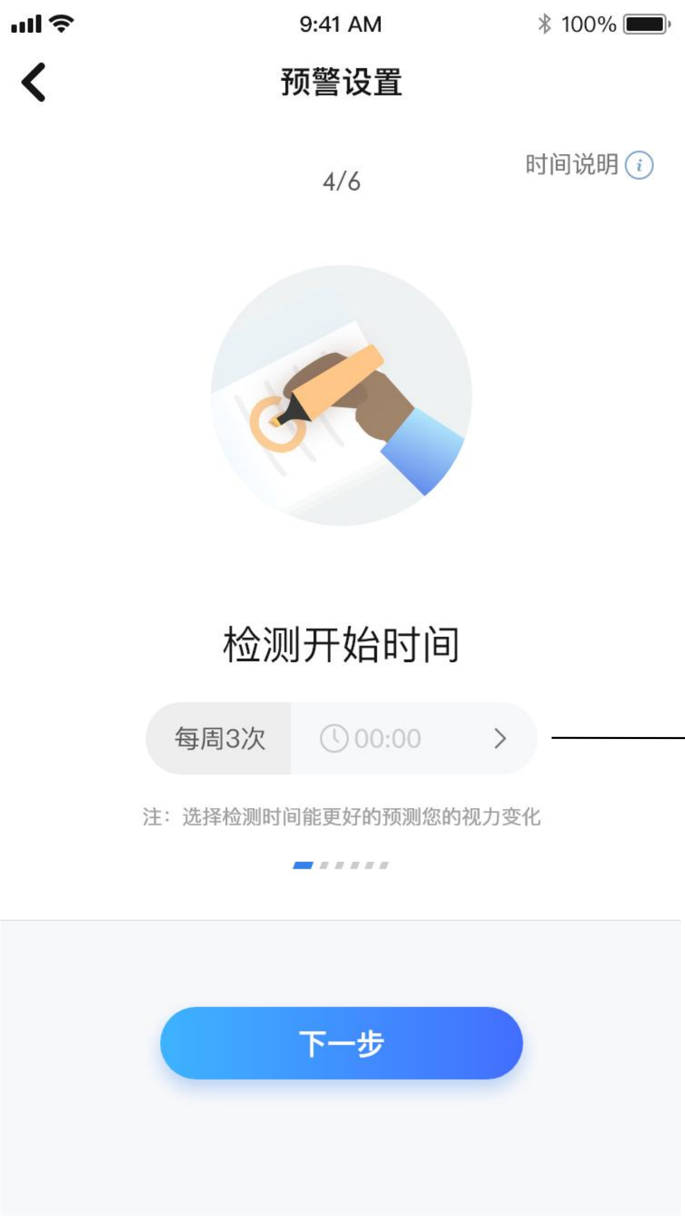

The user configures the monitoring schedule by selecting preferred time intervals for periodic detection sessions.

Figure B-4-1

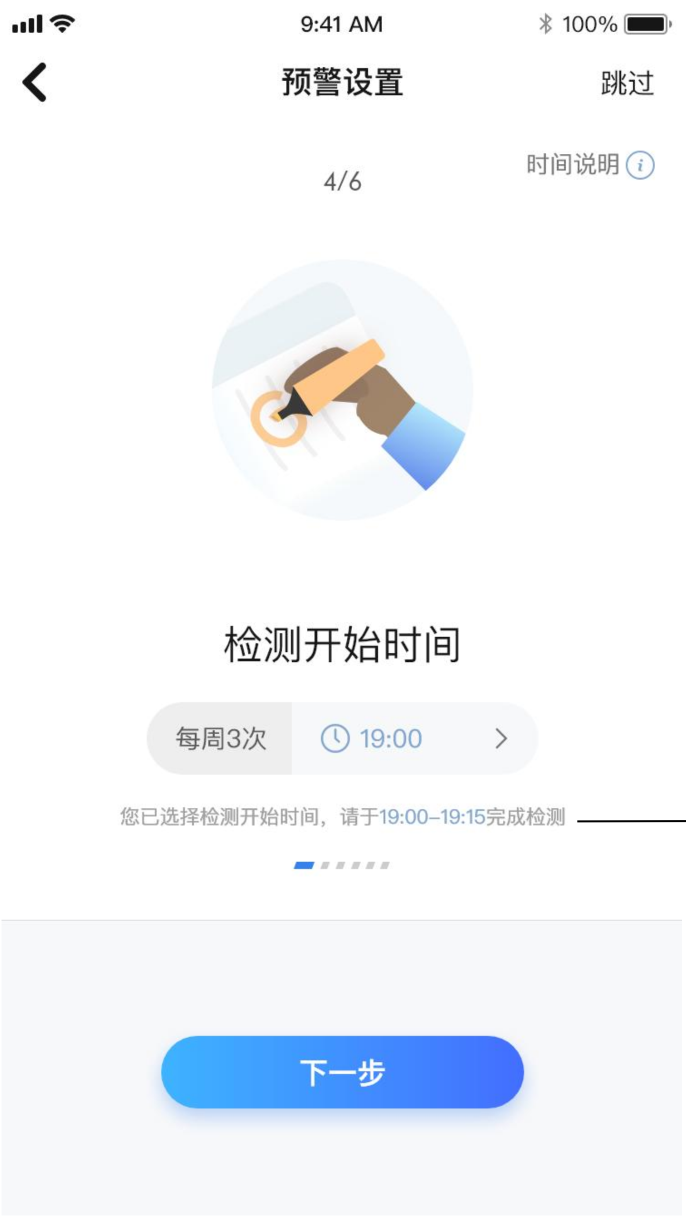

The backend automatically initiates the detection process at scheduled times based on user-configured parameters.

Figure B-4-2

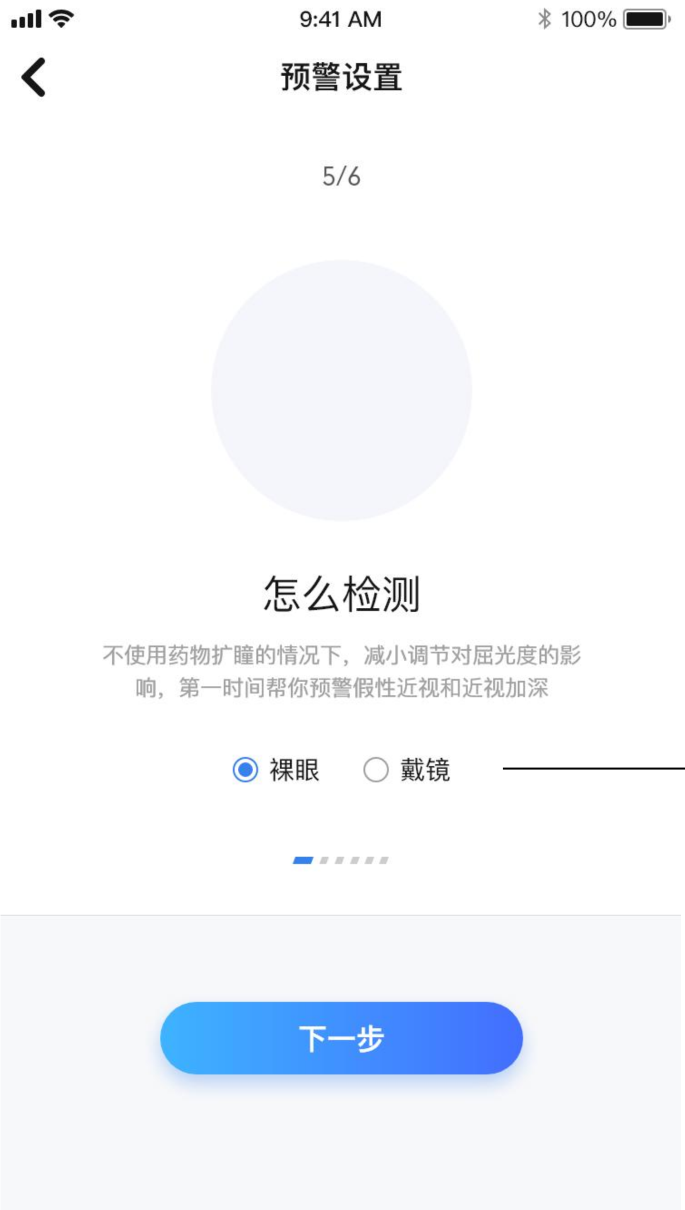

Initially, no option is pre-selected. The user must select either 'Uncorrected (No Lenses)' or 'Corrected (With Lenses)' to activate the Next button and enable navigation to subsequent steps.

Figure B-5

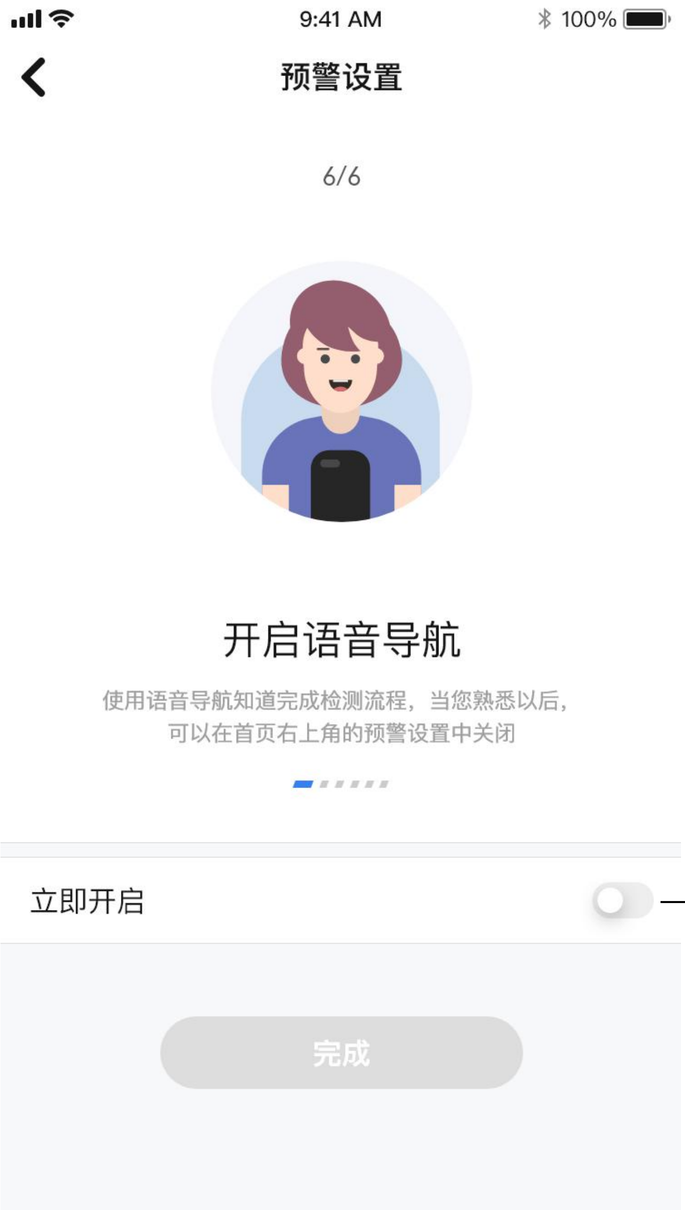

Figure B-6

The voice guidance system defaults to the inactive state. Upon toggling on the voice guidance feature through a click interaction, the completion button becomes enabled (visual indicator: blue highlight). Activation of the completion button triggers transition to the detection initiation interface depicted in Figure C-1.

3. Detection during alert activation

Upon clicking the Complete button in Figure B-6 above, the system immediately navigates to this interface: the backend initiates voice broadcast playback, activates the front-facing camera, instantly transitions to Figure C-2 following broadcast completion, and concurrently enables speech recognition capabilities.

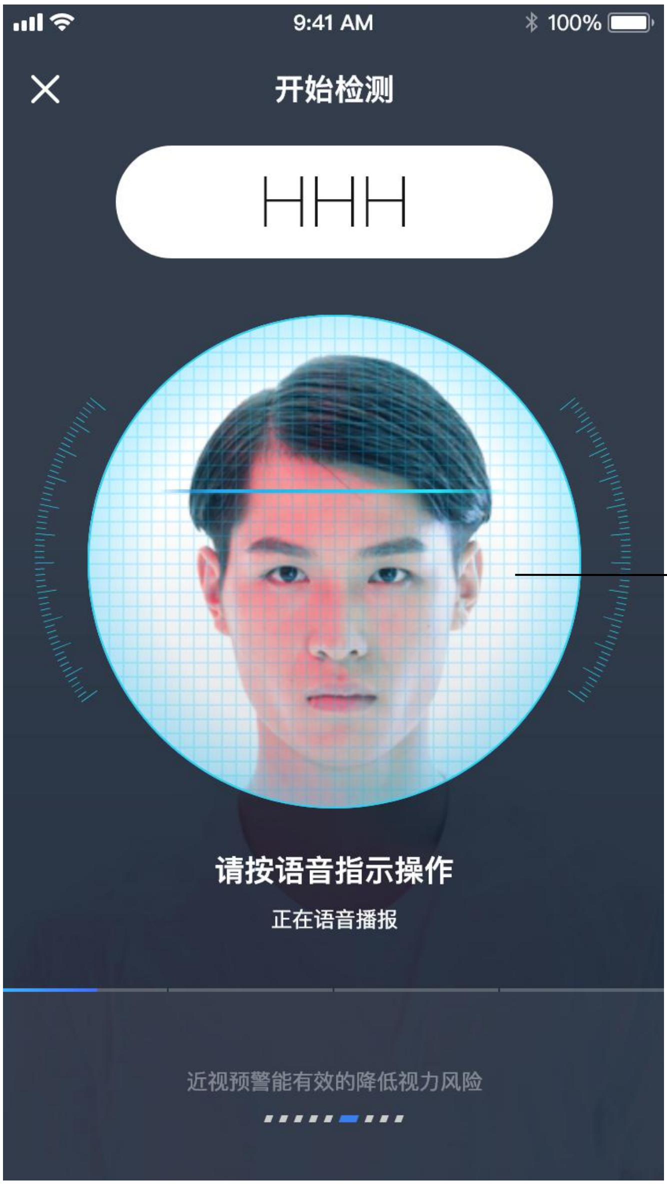

The central facial pattern interface component initiates the front-facing camera's live preview feed upon activation.

Figure C-1

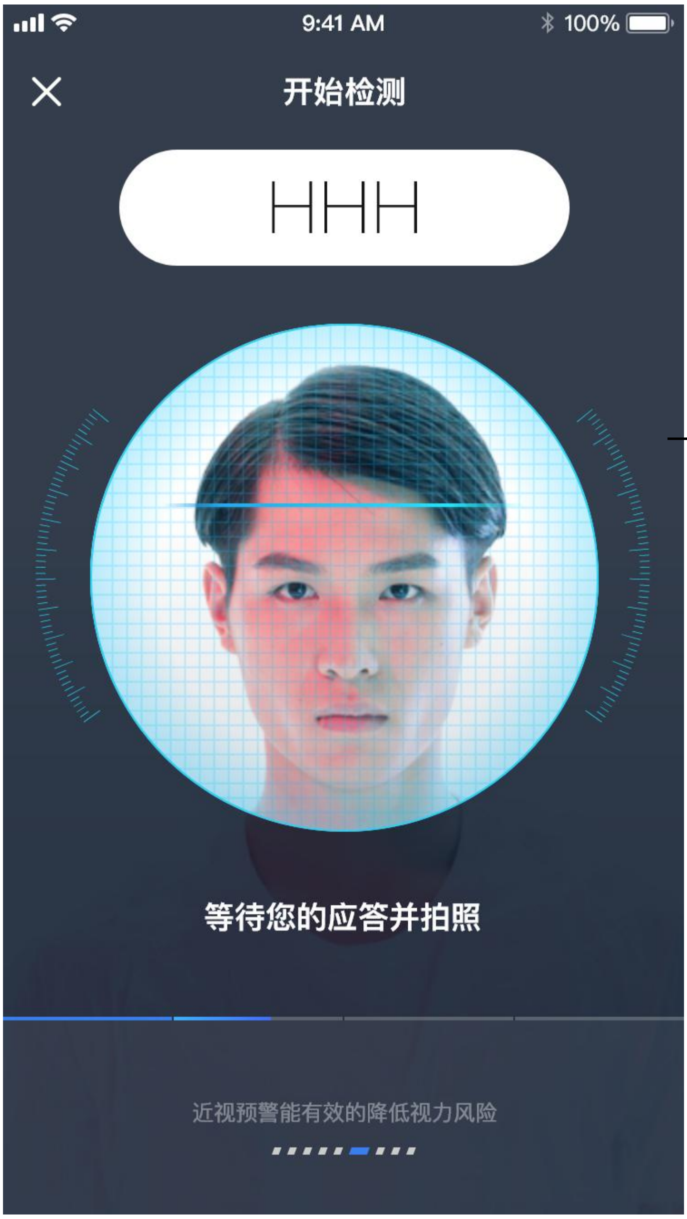

While responding, trigger voice recognition to immediately activate the front camera for photo capture and upload. The front-end displays a camera capture animation on the facial area, then transitions to Figure C-3.

Figure C-2

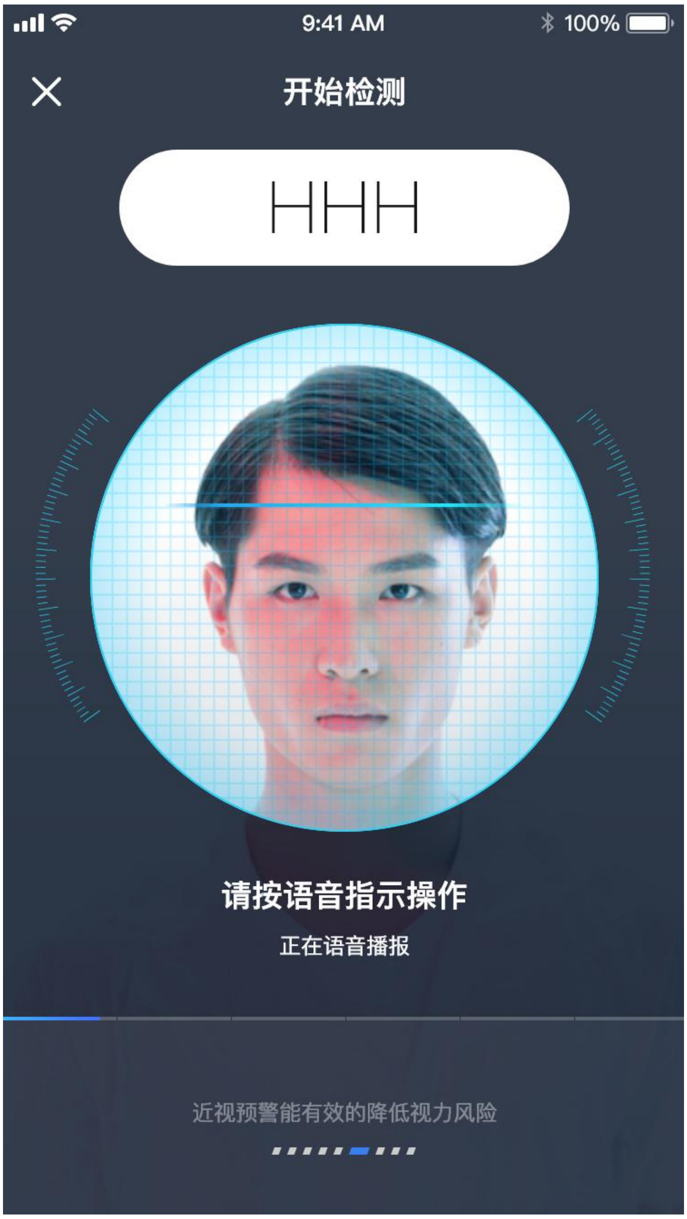

Figure C-3

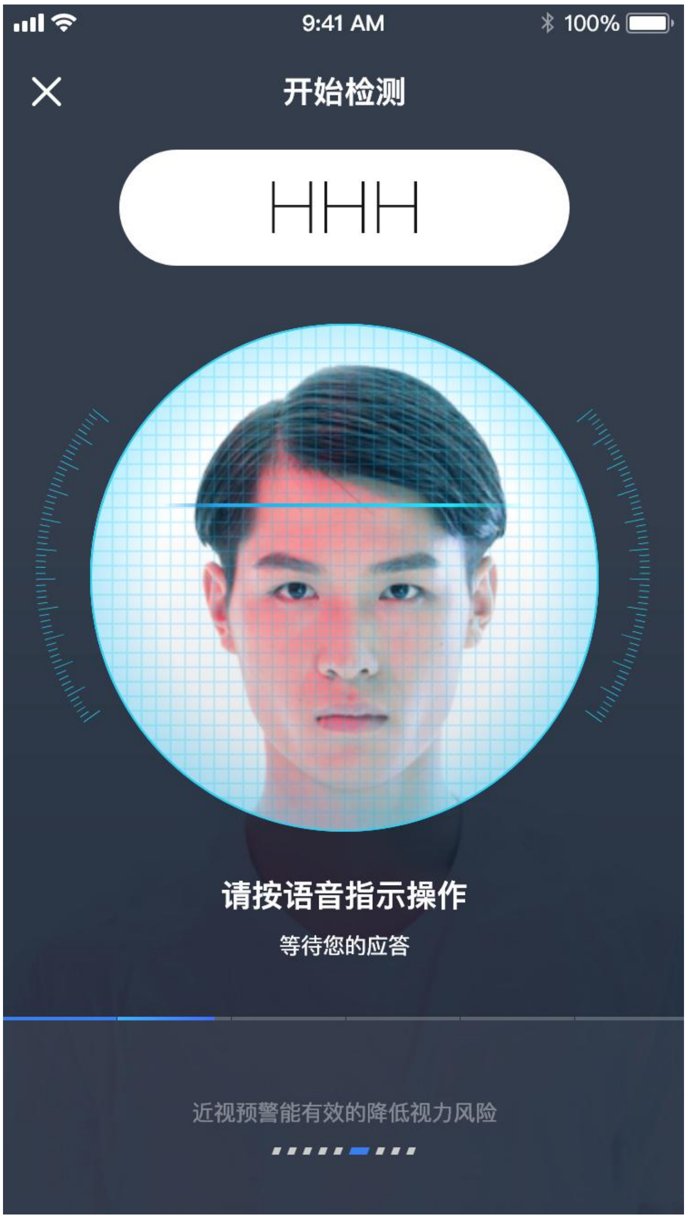

Figure C-4

The process of Figure C-3 and Figure C-4 is to repeat Figure C-1 and Figure C-2, with the only difference being that in C-4, after the user responds, a photo is captured and uploaded, proceeding to Figure C-5.

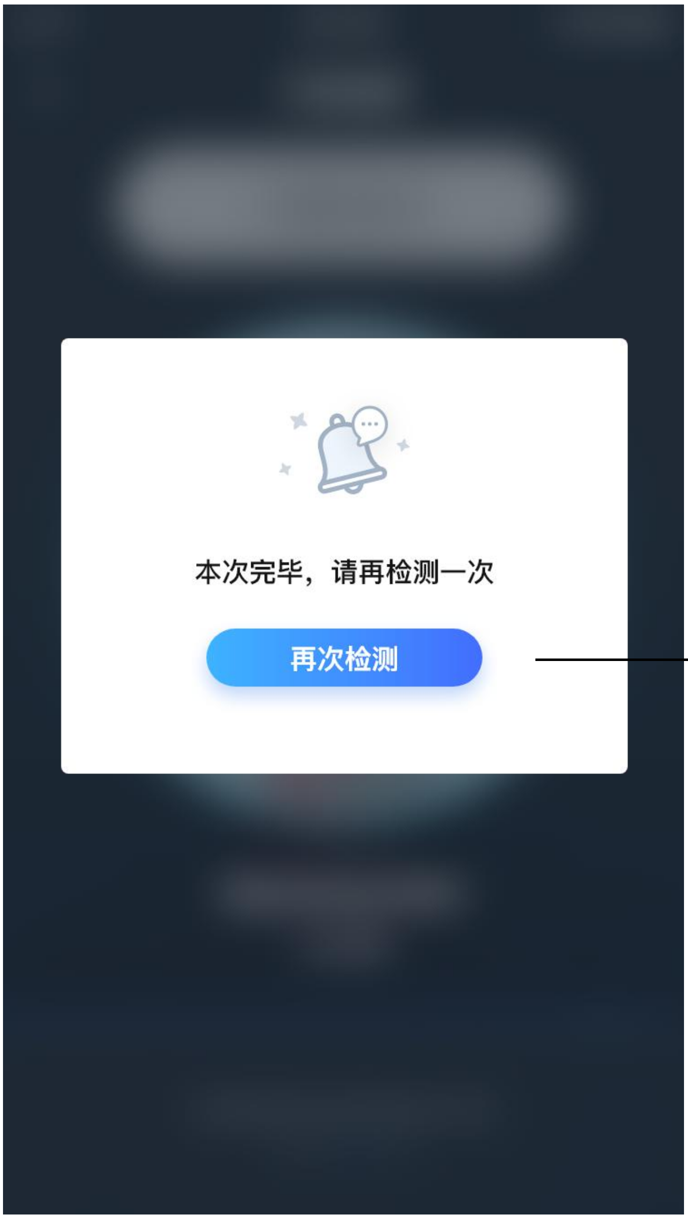

Clicking “Re-detect” or any blank area reinitiates the process flow: C-1→C-2→C-3→C-4→C-6/C-7, where C-6 and C-7 represent successful and unsuccessful outcomes respectively.

Figure C-5

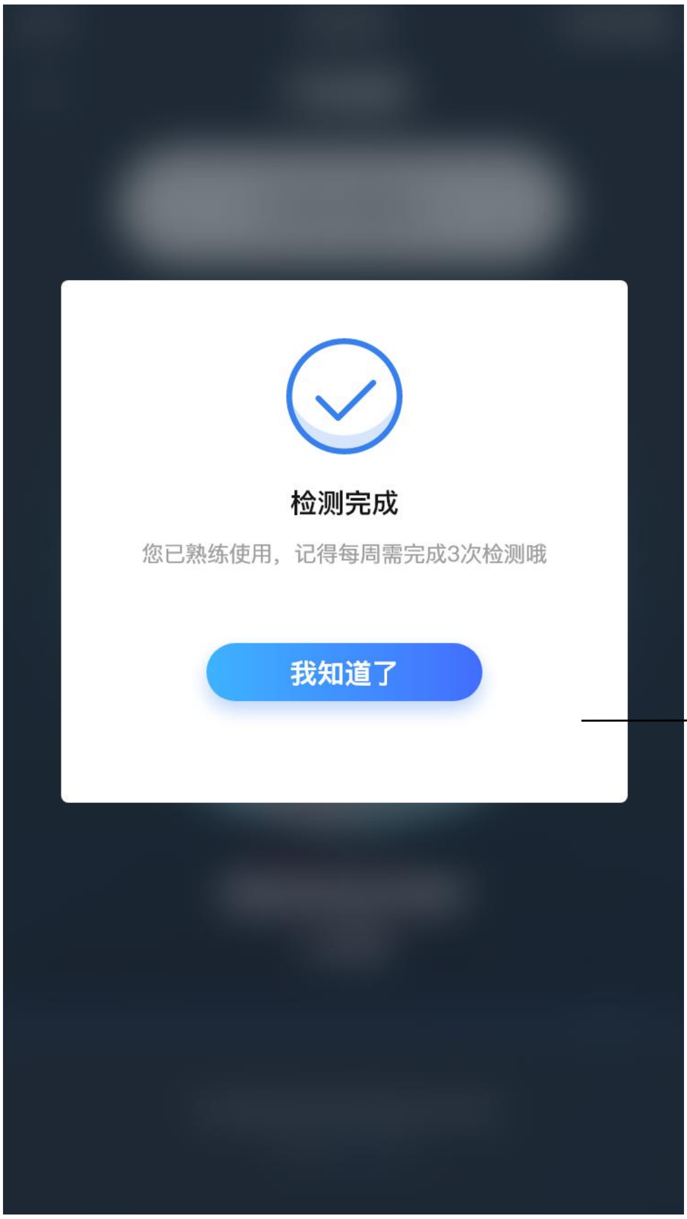

Return to Homepage, Figure A-2

Figure C-6

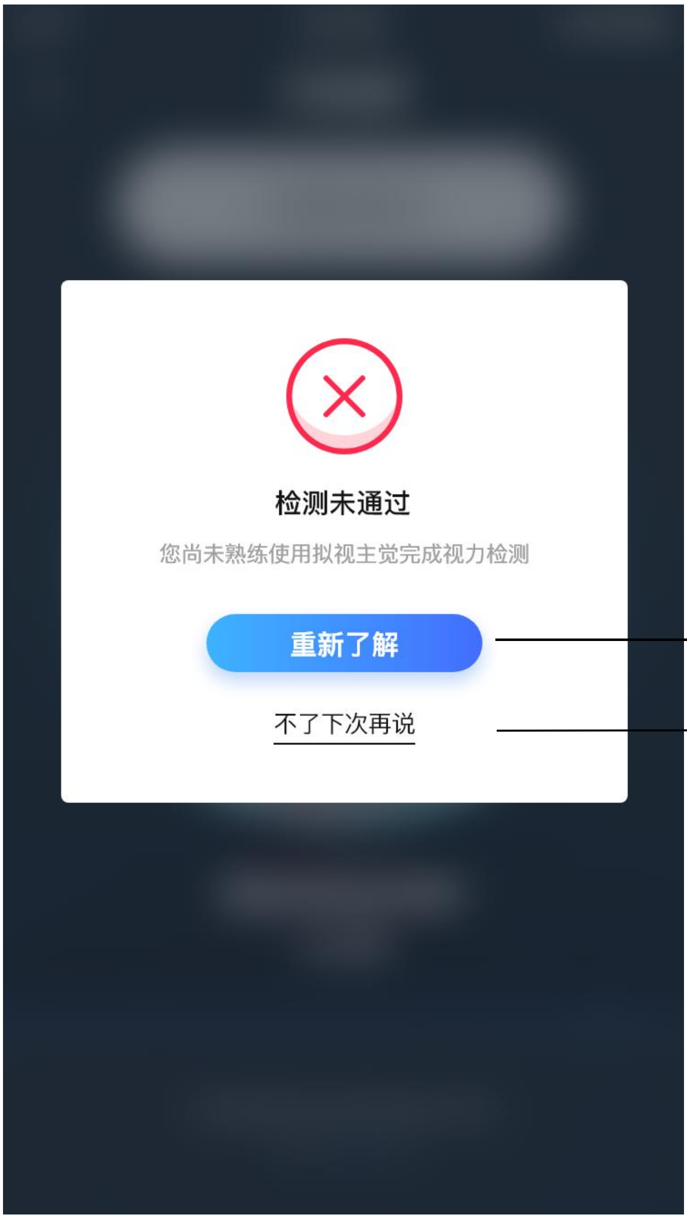

Return to Figure A-1 to restart detection

Return to homepage, Figure A-1

Figure C-7

4. Homepage - Chart

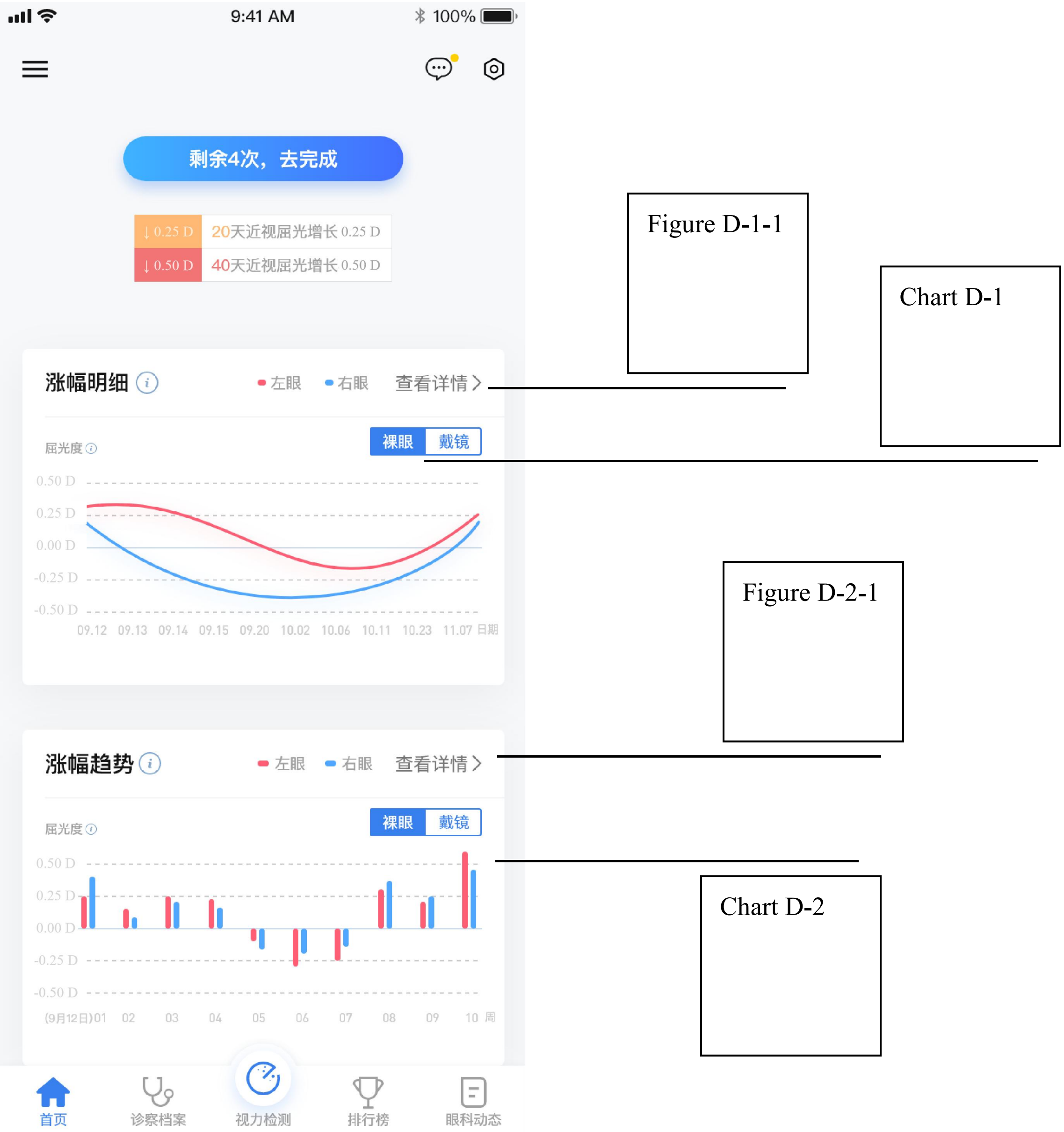

Figure A-1/A-2

Chart D-1

X-axis: Date; Y-axis: -0.50 D, -0.25 D, 0.00 D, 0.25 D, 0.50 D

Chart D-2

X-axis: Week; Y-axis: -0.50 D, -0.25 D, 0.00 D, 0.25 D, 0.50 D

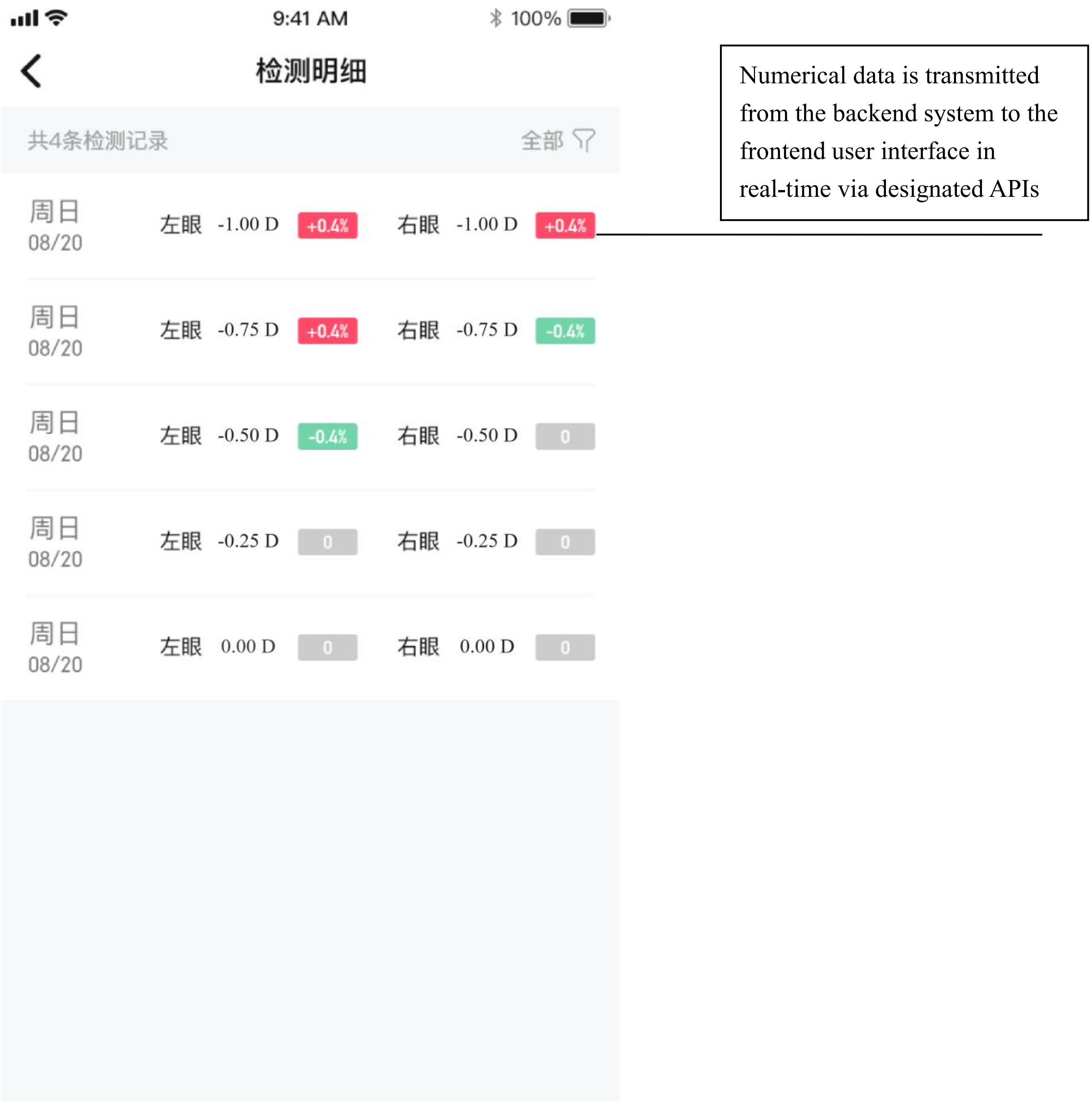

Figure D-1-1/D-2-1

5. Post-alert activation detection

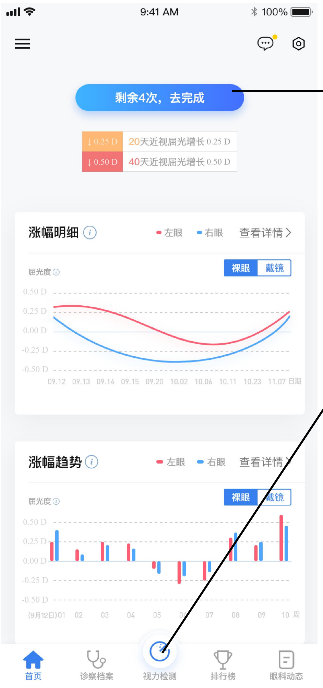

Post-alert activation, initiate detection via these two buttons

Two detection process schemes:
1. Scheme 1: C-1 → C-2 → C-3 → C-4 → C-8/C-11
(Time-delayed return to Homepage A-2 or return via blank area click)
2. Scheme 2: C-9 → C-10 → C-8/C-11
(Time-delayed return to Homepage A-2 or return via blank area click)

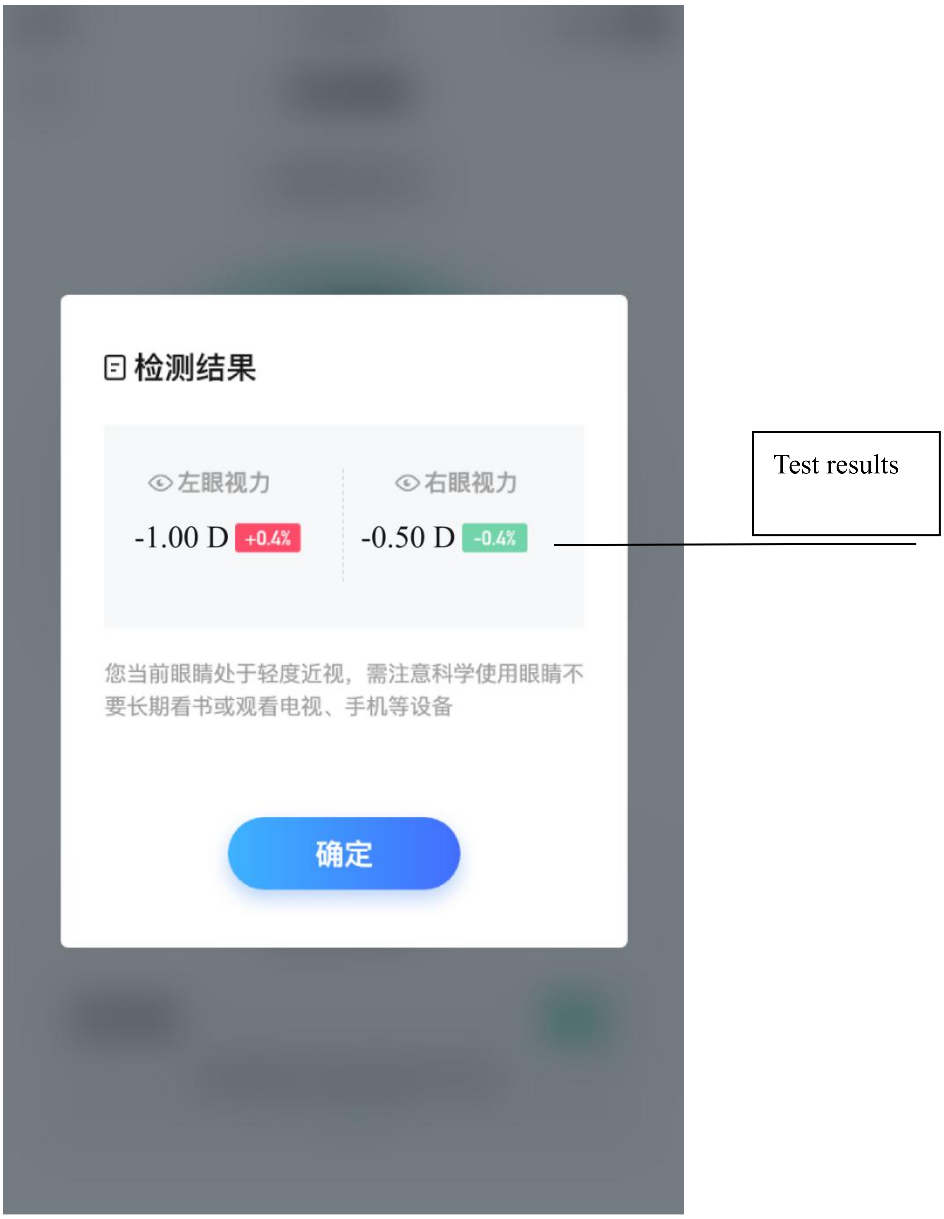
检测结果
左眼视力
-1.00 D +0.4%
右眼视力
-0.50 D -0.4%
您当前眼睛处于轻度近视，需注意科学使用眼睛不要长期看书或观看电视、手机等设备
确定
Test results

Figure C-8

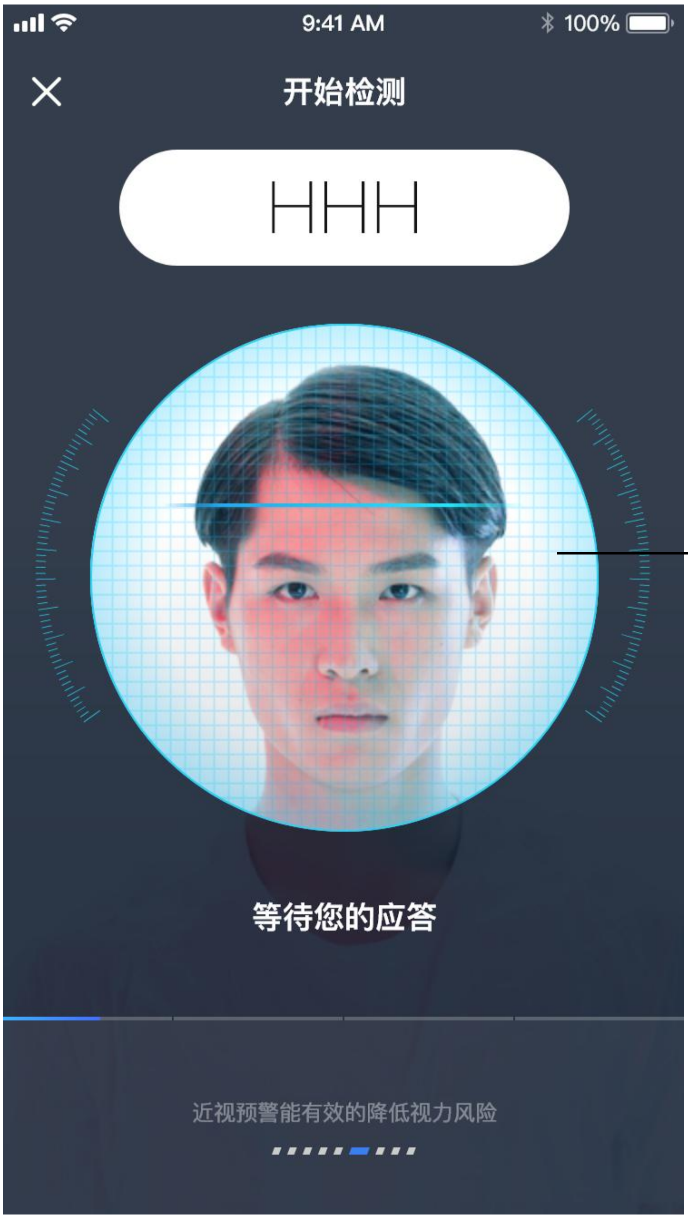

Speech recognition guidance is actively engaged while the system monitors and awaits the user's verbal response input through the audio channel.

Figure C-9

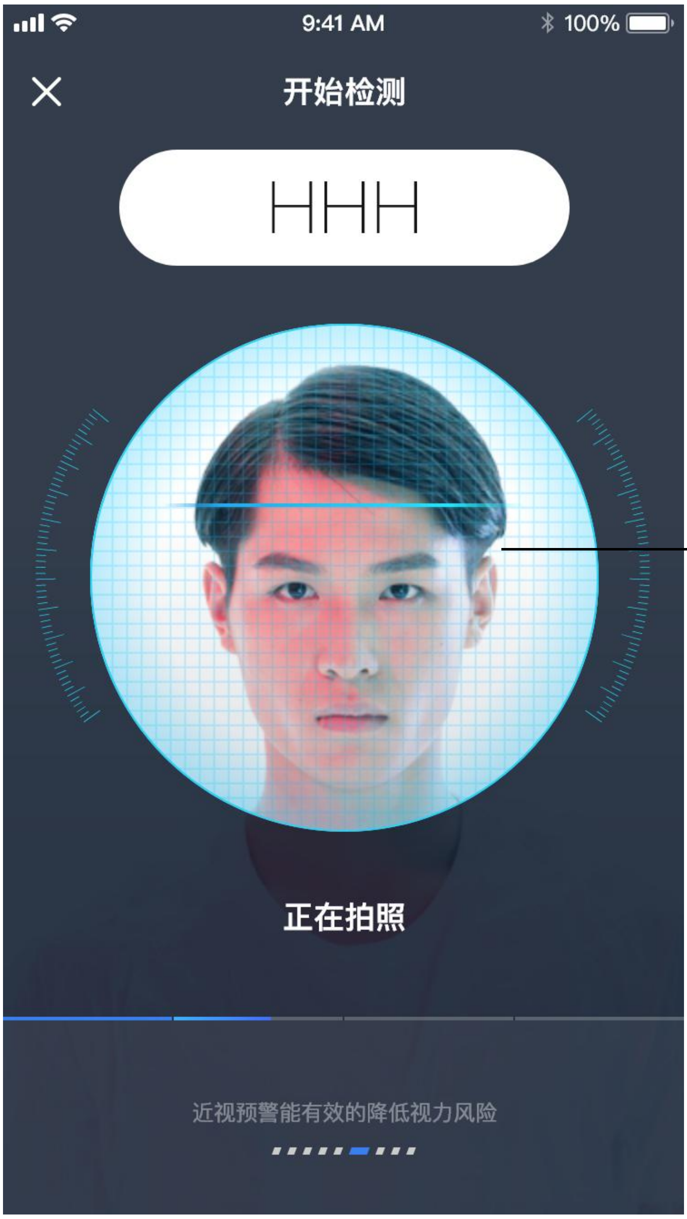

Post-detection completion, the front-facing camera's image acquisition and transmission to cloud storage are activated synchronously upon system validation of the user's verbal response through the speech recognition pipeline.

Figure C-10

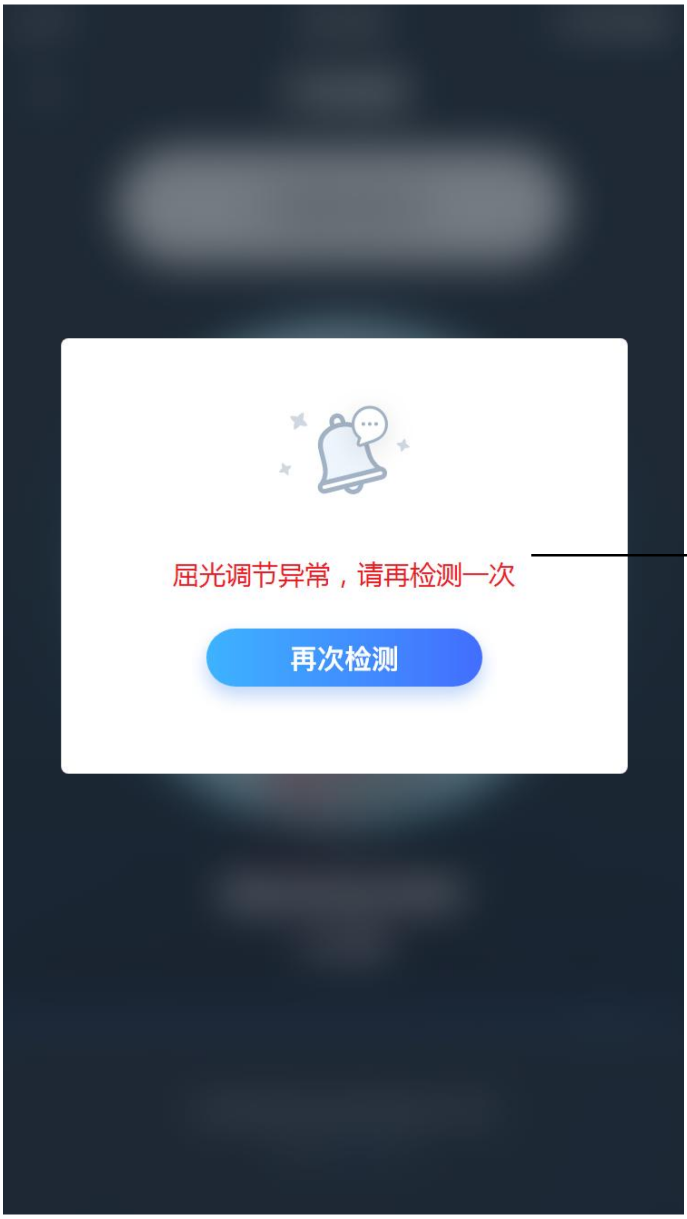

Abnormal refractive results detected, the system requires initiating a secondary detection protocol to reconfirm measurements.

Figure C-11
