# Supplementary material for: Innovative myopic screening platform based on smartphones
Source: Front Bioeng Biotechnol. 2025 Oct 20;13:1678800. doi: 10.3389/fbioe.2025.1678800 (PMC12580630; doi:10.3389/fbioe.2025.1678800)
Supplement: Supplementary file 2 [file DataSheet1.pdf]

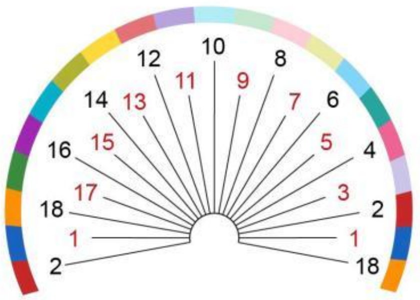

Fig. S1. The schematic diagram of the astigmatism dial.

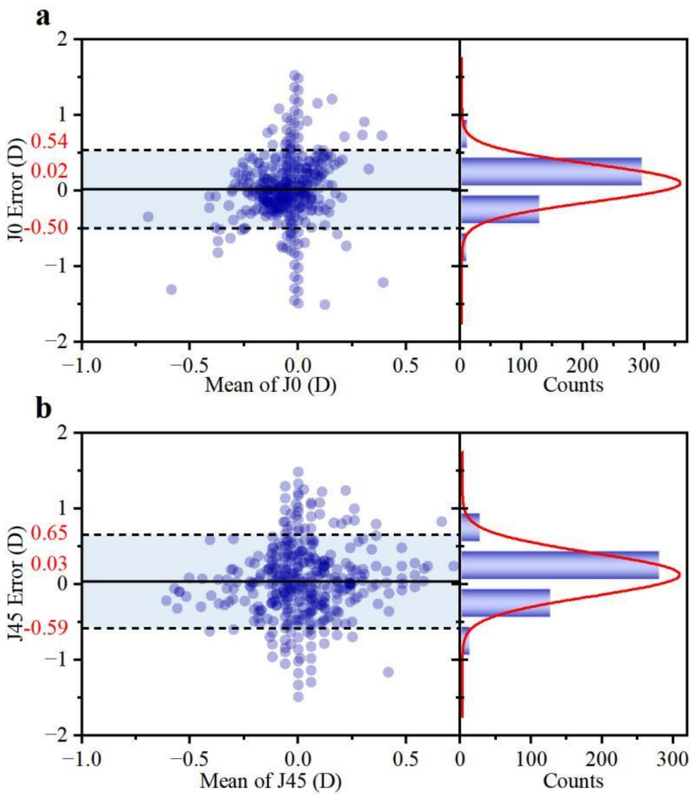

Fig. S2. Bland-Altman analysis of the agreement between the smartphone-based method and subjective refraction for the astigmatic power vector components. The left panel of each figure shows the Bland-Altman plot, while the right panel shows the corresponding Gaussian-fitted histogram of the errors: **(a)** J0 component, **(b)** J45 component.

Table S1. Comparison of difference in SER (D) between teenage and adult groups using independent samples *t*-test.

Features	Adults Group	Teenages Group	*p*-value[2]
n	258	202	
MD[1]	0.06	0.14	>0.05
95% LOAs	0.06 ± 0.92	0.14 ± 0.85	

[1]Mean Difference (MD) calculated as (Smartphone Value - Clinical Value).

[2]*p*-value derived from an independent samples *t*-test. comparing two goups.
